# A heterologous marker-free selection approach for CRISPR/Cas9-based gene editing in the malaria parasite *Plasmodium falciparum*

**DOI:** 10.1128/msphere.00884-25

**Published:** 2026-03-26

**Authors:** Eilidh Carrington, Daniel Ballmer, Igor Niederwieser, Basil T. Thommen, Nicolas M. B. Brancucci, Till S. Voss

**Affiliations:** 1 Department of Medical Parasitology and Infection Biology, Swiss Tropical and Public Health Institute 30247, Allschwil, Switzerland; 2 University of Basel 27209, Basel, Switzerland; Australian National University, Canberra, ACT, Australia

**Keywords:** malaria, *Plasmodium falciparum*, CRISPR/Cas9

## Abstract

CRISPR/Cas9-based gene editing of the malaria parasite *Plasmodium falciparum* has emerged as a transformative tool for advancing functional studies on parasite biology and identifying new therapeutic targets. Currently applied CRISPR/Cas9 methodologies depend on a limited set of heterologous drug resistance markers for the selection of transgenic parasites, which restricts the potential for iterative genetic modifications. Here, we developed a heterologous marker-free CRISPR/Cas9 gene editing strategy (CRISPR/Cas9^pyrR^) for *P. falciparum* based on the simultaneous editing of a gene of interest and introduction of pyrimethamine (PYR) resistance-conferring mutations into the dihydrofolate reductase-thymidylate synthase (*pfdhfr-ts*) gene. By providing a *pfdhfr^pyrR^* donor sequence and the Cas9 expression cassette on separate plasmids, CRISPR/Cas9^pyrR^ ensures that only parasites acquiring both plasmids survive under PYR pressure. As a proof of principle, we applied CRISPR/Cas9^pyrR^ to generate two transgenic parasite lines expressing GFP-tagged versions of the putative nuclear envelope protein PfGEX1 and nuclear pore protein PfNUP116, respectively. We show that PfGEX1-GFP marks the nuclear envelope specifically in gametocytes, but not in asexual blood stage parasites. Similarly, and against previous reports, we find PfNUP116-GFP expression is undetectable in asexual parasites but instead localizes to a distinct perinuclear region in early gametocytes. These results suggest dynamic compositional changes of the nuclear periphery during sexual differentiation. We further demonstrate sequential genetic engineering of the PfNUP116-GFP-expressing line using the human *dhfr* drug resistance marker combined with WR99210-based selection by additionally tagging PfAP2-G, the master transcriptional regulator of sexual commitment, and the nuclear pore protein PfNUP313. Hence, CRISPR/Cas9^pyrR^ provides a versatile and effective new method that enhances and complements the current genetic toolkit for malaria research.

**IMPORTANCE:**

Malaria tropica, which is caused by the unicellular parasite *Plasmodium falciparum*, is one of the most devastating infectious diseases worldwide. The development of urgently needed effective vaccines and new antimalarial drugs with novel modes of action requires a profound understanding of parasite biology. CRISPR/Cas9-based genome engineering is beyond doubt the most important experimental approach to study the function and essentiality of parasite proteins and to identify and validate new vaccine and drug targets. In this study, we developed and successfully applied a modified CRISPR/Cas9 strategy, termed CRISPR/Cas9^pyrR^, that avoids the use of a heterologous drug resistance marker for the selection of genetically modified parasites. CRISPR/Cas9^pyrR^ thus complements the CRISPR/Cas9 toolbox available for gene editing in *P. falciparum* and overcomes some of the limitations of currently employed protocols.

## INTRODUCTION

Hundreds of millions of people are infected with *Plasmodium falciparum*, an obligate intracellular protozoan parasite of the phylum *Apicomplexa* and the causative agent of the most severe form of malaria in humans. This devastating disease caused over 250 million clinical cases and 600,000 deaths globally in 2023, predominantly among young children and pregnant women in sub-Saharan Africa (1). Approximately 90% of malaria cases and fatalities occur in this region. The rapid emergence and spread of parasite strains resistant to antimalarial drugs emphasize the critical need for new therapeutics and vaccines, the development of which hinges on a detailed understanding of the molecular mechanisms governing parasite biology (2).

*P. falciparum* has a complex life cycle with multiple parasite stages developing in the human host and the female *Anopheles* spp. mosquito vector. When taking a blood meal, the infected mosquito injects sporozoites into the skin from where they enter circulation. After a first round of parasite multiplication inside hepatocytes, infected liver cells release thousands of merozoites into the blood stream, marking the onset of the pathogenic phase of the infection. Here, *P. falciparum* undergoes vegetative growth via repeated rounds of red blood cell (RBC) invasion and intra-erythrocytic replication. After merozoite invasion, the parasite develops through the ring (0–24 hours post invasion [hpi]) and trophozoite stage (24–30 hpi) before it replicates via schizogony (30–48 hpi), ultimately releasing 20–32 daughter merozoites ready to invade new RBCs. During each intra-erythrocytic cycle, a small proportion of schizonts produce sexually committed ring stage progeny that differentiates over the course of 10–12 days into mature female or male gametocytes. Early gametocyte stages sequester in the bone marrow and spleen parenchyma of the human host, where they develop through four morphologically distinct stages (I–IV) before the crescent-shaped stage V forms re-enter circulation (3–5). When ingested by a blood-feeding mosquito, stage V gametocytes transform into gametes that undergo sexual reproduction in the mosquito midgut before sporogony eventually gives rise to a new generation of sporozoites capable of infecting another human host.

Although the *P. falciparum* genome was sequenced over 20 years ago, many parasite genes remain functionally unannotated. CRISPR/Cas9-based genetic engineering has emerged as an invaluable tool for studying gene function, uncovering therapeutic targets, or developing genetically attenuated parasites for vaccine development (6, 7). Because *P. falciparum* blood stage parasites are haploid and lack a canonical non-homologous end-joining repair pathway (8, 9), the Cas9-induced double strand break (DSB) can only efficiently be repaired through a specific donor sequence serving as a template for homology-directed repair (HDR) (6). Hence, for successful gene editing in *P. falciparum*, four key components must be introduced into the same parasite: (i) the Cas9 endonuclease; (ii) a single-guide RNA (sgRNA) specific for the target locus; (iii) a donor DNA template containing the desired genetic modification(s) flanked by sequences for HDR; and (iv) one or more heterologous drug resistance markers facilitating the selection of modified parasites. These components are often delivered through transfection of two separate plasmids—the first encoding the Cas9 nuclease and a drug-selectable marker, and the second (so-called ‘donor’ plasmid) providing the sgRNA, homologous regions for recombination and an additional drug-selectable marker (10–14).

Despite CRISPR/Cas9 gene editing approaches having frequently been applied and further adapted for the use in *P. falciparum* for over a decade, genome engineering in this parasite remains a challenge. In addition to low transfection efficiencies (15, 16), the limited repertoire of heterologous drug resistance markers available for malaria parasites (human dihydrofolate reductase [hDHFR; confers resistance to WR99210], *Aspergillus terreus* blasticidin deaminase [AtBSD; confers resistance to BSD-S-HCl], yeast dihydroorotate dehydrogenase [yDHODH; confers resistance to DSM-1]) restricts the ability to perform sequential genetic modifications. Furthermore, BSD-S-HCl and DSM-1 are not entirely reliable, as parasites can gain resistance to these drugs via an epigenetic switch in the expression of *clag3* paralogs (17) or amplification of the *pfdhodh* gene (18), respectively. An improved strategy initially described by Lu et al. (19), and then also by us (20) and other labs (21, 22), addresses this limitation using a ‘suicide-rescue’-based approach. This method eliminates the need for a second drug resistance marker by employing a ‘suicide’ vector that provides the Cas9 enzyme, sgRNA, and a heterologous drug selection marker, alongside a ‘rescue’ construct containing the donor DNA template without any selectable marker. Using this strategy, only parasites that successfully take up both plasmids and succeed in repairing the Cas9-induced DSB by HDR using the provided donor DNA template survive under drug selection. However, this approach nevertheless relies on a heterologous drug-selectable marker that may persist episomally within the modified parasite strain and impede downstream applications (19, 23). While the addition of negative selection markers has been applied for episomal plasmid elimination (10, 12, 21, 23, 24), this strategy prolongs the selection process and is not always 100% efficient (25, 26). Other studies employed so-called “all-in-one” plasmids that contain expression cassettes for Cas9, sgRNA, a heterologous drug-selectable marker as well as the donor template, but the substantially larger size of these constructs complicates molecular cloning (20, 22, 27–29).

To overcome some of these challenges, we developed a two-plasmid CRISPR/Cas9-based gene editing approach for *P. falciparum* that does not rely on the use of heterologous drug resistance markers to select for genetically modified parasites. Instead, along with one plasmid delivering the Cas9 expression cassette and a sgRNA and donor template targeting the gene of interest (GOI), we co-transfect a second plasmid providing a sgRNA and donor sequence designed to insert mutations into the parasite dihydrofolate reductase-thymidilate synthase gene (*pfdhfr-ts*) that confer resistance to the antimalarial antifolate drug pyrimethamine (PYR). We successfully implement this approach, called CRISPR/Cas9^pyrR^, for the C-terminal tagging of two putative components of the nuclear envelope (NE), PfGEX1 and PfNUP116, and describe their localization dynamics throughout the asexual intra-erythrocytic development cycle (IDC) and gametocyte differentiation. We also demonstrate that CRISPR/Cas9^pyrR^-engineered parasites remain sensitive to the antifolate WR99210 and are hence amenable to sequential engineering using human *dhfr* (h*dhfr*) as a heterologous drug-selectable marker.

## RESULTS

### Design of the CRISPR/Cas9^pyrR^ method for marker-free gene editing in *Plasmodium falciparum*

The antimalarial drug PYR is a folate antagonist that inhibits the essential bi-functional parasite enzyme PfDHFR-TS and has potent activity against *P. falciparum* schizonts, killing parasites *in vitro* with half-maximal inhibitory concentrations (IC_50_) in the low to single-digit nanomolar range (30, 31). In the 1990s, PYR (as a combination drug with sulfadoxine) served as a first-line treatment for malaria in sub-Saharan Africa but was gradually replaced by artemisinin-based combinations after the rapid emergence and worldwide spread of PYR-resistant *P. falciparum* strains (31–38). A single amino acid change in the active site of the PfDHFR moiety (S108N) increases parasite tolerance to PYR by about two orders of magnitude, and ancillary mutations (N51I, C59R, I164L) cause high-level PYR resistance (*in vitro* IC_50_ > 5 µM) (30, 31, 39–41). Interestingly, PfDHFR-TS mutants (S108N single mutant or N51I, C59R, S108N triple mutant) were the first drug resistance markers used to select for transgenic *P. falciparum* parasites transfected with plasmid DNA (42).

**Fig 1:**
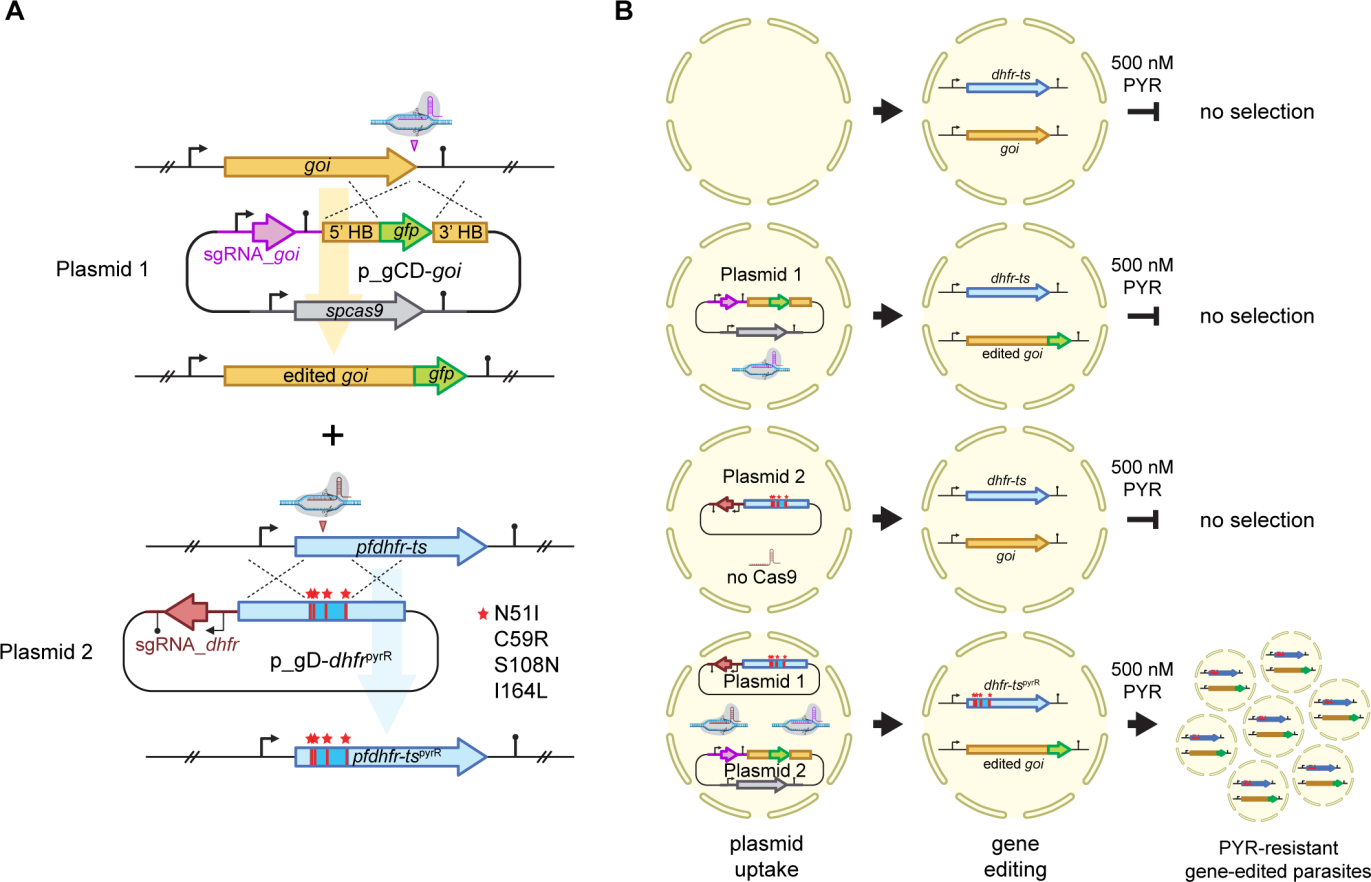
Schematic illustration of the CRISPR/Cas9^pyrR^ approach. (**A**) CRISPR/Cas9^pyrR^ involves the co-transfection of two plasmids: Plasmid 1 (p_gCD_*goi*) contains an expression cassette for SpCas9, a single-guide RNA (sgRNA) targeting a gene of interest (sgRNA_*goi*), and a donor template tailored to modify the gene of interest as desired. Plasmid 2 (p_gD-*dhfr*^pyrR^) encodes a sgRNA targeting the *pfdhfr-ts* gene (sgRNA-*dhfr*) along with the *dhfr^pyrR^* donor template that introduces four specific point mutations into the *pfdhfr-ts* coding region. These mutations result in a highly PYR-resistant PfDHFR-TS variant (PfDHFR-TS^pyrR^; N51I, C59R, S108N, I164L) and are indicated by red asterisks. The central re-codonized region of the *dhfr^pyrR^* donor sequence encompassing the four mutations is shaded in bright blue. The relative positions of the sgRNA target sites are indicated by vertical arrowheads. (**B**) Expected outcomes of transfection after the uptake of either plasmid 1 alone, plasmid 2 alone, or both plasmids together. Parasites that take up plasmid 1 alone will not survive PYR challenge because they express wild-type PfDHFR-TS. Parasites receiving plasmid 2 alone will also be sensitive to PYR because editing of the *pfdhfr-ts* gene using the *dhfr^pyrR^* donor template is unlikely to occur in the absence of SpCas9 expression. Hence, only parasites receiving both plasmids can simultaneously edit both loci and survive in the presence of 500 nM PYR.

We reasoned that the simple genetic mechanism underlying PYR resistance may be co-opted to develop a CRISPR/Cas9-based gene editing strategy for *P. falciparum* that obviates the need for heterologous drug resistance markers for the selection of transgenic parasites. This strategy is grounded in the co-transfection of two plasmids. Plasmid 1 (p_gCD_*goi*) encodes an expression cassette for *Streptococcus pyogenes* Cas9 (SpCas9), an expression cassette for a sgRNA targeting the gene of interest (sgRNA_*goi*) and a donor template designed as needed to modify the target gene via HDR of the DSB. Plasmid 2 (p_gD-*dhfr*^pyrR^) carries an expression cassette for a sgRNA targeting the *pfdhfr-ts* gene (sgRNA-*dhfr*) and a partially re-codonized donor template to introduce four point mutations into the *pfdhfr-ts* coding sequence that will result in expression of a highly PYR-resistant PfDHFR-TS quadruple mutant (PfDHFR-TS^pyrR^; N51I, C59R, S108N, I164L) (Fig. 1A; Fig. S1). While plasmid 1 carries all components necessary for CRISPR/Cas9-based editing of the gene of interest, parasites receiving plasmid 1 alone will not survive PYR challenge because they express wild-type PfDHFR-TS. Parasites receiving plasmid 2 alone will also be sensitive to PYR because insertion of the PYR resistance-conferring mutations provided by the *dhfr^pyrR^* donor template will not occur in the absence of SpCas9 expression. Hence, the only parasites able to survive and propagate in the presence of PYR are those receiving both plasmids simultaneously (Fig. 1B).

### Application of the CRISPR/Cas9^pyrR^ method for C-terminal tagging of PfGEX1

As proof of concept, we first employed CRISPR/Cas9^pyrR^ to engineer NF54 parasites expressing a GFP-tagged version of the putative NE-associated protein PfGEX1 (PF3D7_1363800). The NE is a double-membrane structure embedded with nuclear pore complexes (NPCs) that facilitates selective molecular exchange between the nucleus and the cytoplasm. Beyond its structural and transport functions, the NE in malaria parasites plays a crucial role in mitosis by housing the centriolar plaque, a specialized microtubule organizing center (MTOC) that supports spindle formation, kinetochore-microtubule attachments, and chromosome segregation (43–45). Despite the importance of the NE for parasite survival and multiplication, its associated protein inventory in *P. falciparum* remains poorly characterized.

**Fig 2:**
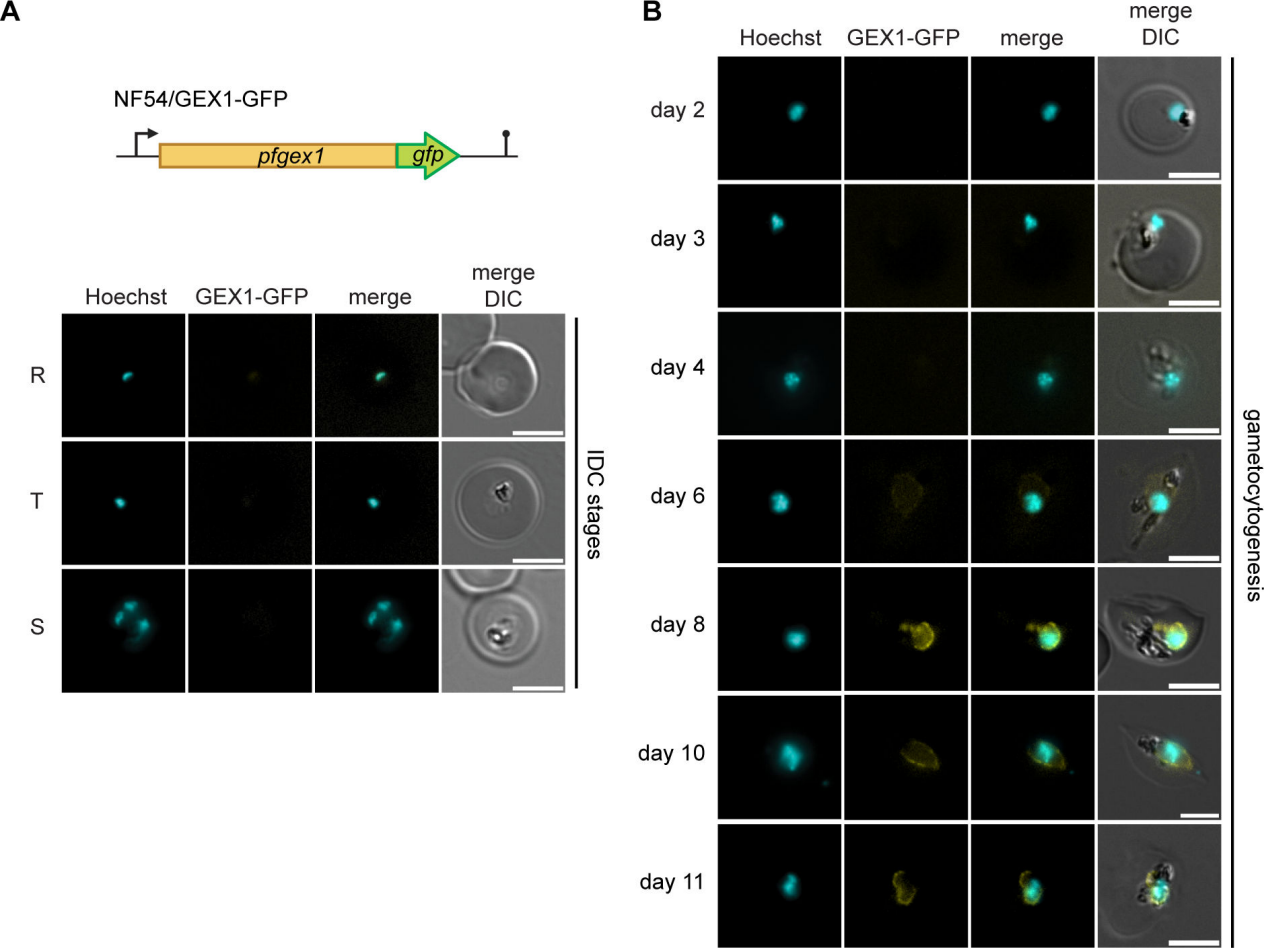
PfGEX1 marks the nuclear envelope in late-stage gametocytes. (**A**) Top: schematic illustration of the modified *pfgex1* locus in NF54/GEX1-GFP parasites. Bottom: representative live cell fluorescence images of ring stage (R), trophozoites (T), and schizonts (S) showing lack of PfGEX1-GFP expression during the asexual intra-erythrocytic development cycle (IDC). (**B**) Representative live cell fluorescence images of gametocytes throughout sexual development (days 2–11) showing PfGEX1-GFP expression in stage III–V gametocytes (days 6–11). DNA was stained with Hoechst. DIC, differential interference contrast. Scale bar, 5 μm.

GEX1 proteins were recently identified as members of an ancient eukaryotic KAR5/GEX1 family comprising NE-linked proteins involved in sexual reproduction across diverse eukaryotic taxa (46). In *P. berghei*, PbGEX1 localizes to the NE in gametocytes and is essential for producing viable meiotic progeny (46, 47). The *pfgex1* ortholog in *P. falciparum* shows gametocyte-specific transcription (48–50), but PfGEX1 localization to the NE has yet to be confirmed. To investigate PfGEX1 localization dynamics in *P. falciparum*, we co-transfected NF54 parasites with the CRISPR/Cas9 plasmid p_gCD-*gex1-gfp* and the donor plasmid p_gD-*dhfr*^pyrR^ (Fig. 2A; Fig. S2). After constant exposure to PYR, we observed a stably propagating population of PYR-resistant parasites within approximately 3 weeks after transfection. Following limiting dilution cloning, we obtained five clonal lines that all carried a successfully modified *pfgex1* locus (Fig. S2). NF54/GEX1-GFP clone B7 was selected for further studies and diagnostic PCRs on genomic DNA (gDNA) confirmed the correct editing of both the *pfgex1* and *pfdhfr-ts* loci and the absence of wild-type parasites (Fig. S2). Additionally, Sanger sequencing of the *pfdhfr-ts* gene verified the presence of the PYR-resistance conferring mutations in these parasites (Fig. S2). Interestingly, we observed that the 3′ crossover event between the endogenous *pfdhfr-ts* gene and *dhfr^pyrR^* donor template did not occur via the 3′ homology box but rather took place in the region located between the S108N and I164L codons. As a result, only the first three of the four PYR resistance-conferring point mutations (N51I, C59R, S108N) have been introduced, consistent with previous reports showing that this triple mutant is also highly resistant to PYR (42).

PfGEX1-GFP expression was undetectable in asexual blood stage parasites as assessed by live cell fluorescence microscopy (Fig. 2A). To assess PfGEX1-GFP expression and localization over the course of gametocyte development, we induced sexual commitment in synchronous NF54/GEX1-GFP parasites using minimal fatty acid (mFA) medium (51) and maintained the resulting progeny (day 1 of gametocyte differentiation) in serum-containing medium, supplemented with 50 mM N-acetylglucosamine (GlcNAc) for the first 6 days to eliminate asexual parasites (52). PfGEX1-GFP signals first became apparent as a faint perinuclear band surrounding the Hoechst-stained nuclear DNA in day 6 gametocytes (stage III) and remained enriched at the NE thereafter, with peak expression observed at days 8–9 of gametocyte development (stage IV/V) (Fig. 2B). Consistent with previous reports (24, 53–55), gametocyte nuclei as outlined by PfGEX1-GFP adopted complex morphologies, involving elongated and lobed structures extending beyond the Hoechst-stained region of the nucleus. These findings establish PfGEX1 as a suitable marker of the NE in *P. falciparum* late-stage gametocytes, providing a valuable tool to further explore the nuclear morphological changes during gametocyte differentiation and their functional significance.

### Application of the CRISPR/Cas9^pyrR^ method for C-terminal tagging of PfNUP116

To further validate CRISPR-Cas9^pyrR^ as a genome editing strategy in *P. falciparum*, we turned our attention to PfNUP116 (PF3D7_1473700), one of seven putative nuclear pore proteins (NUP100/221, NUP116, NUP138, NUP205, NUP313, NUP637; SEC13) that have been identified bioinformatically and reported to localize to the perinuclear region in *P. berghei* and/or *P. falciparum* (45, 56–63). Based on immunofluorescence assays (IFAs) using an antibody raised against a synthetic PfNUP116-derived peptide, Lopez-Rubio et al. initially reported PfNUP116 localization as punctate signals along the nuclear periphery across all asexual stages of the IDC (63). In contradiction with these observations, more recent transcriptomics analyses indicate that *pfnup116* is not expressed (or only expressed at very low levels) during the IDC and is instead activated in sexual ring stage parasites (20, 50, 64, 65), hinting at the possibility that PfNUP116 may represent a gametocyte-specific NPC component in *P. falciparum*.

To explore this possibility, we used CRISPR/Cas9^pyrR^ to tag PfNUP116 in NF54 parasites with GFP fused to an FKBP destabilization domain (DD) (NF54/NUP116-GFPDD) (Fig. S3). The FKBP/DD system enables protein destabilization upon withdrawal of the small molecule ligand Shield-1 (66). After the PYR-based selection of transfected parasites and limiting dilution cloning, we obtained 10 clonal lines, of which five each carried a successfully modified or unedited *pfnup116* locus (Fig. S3). PCRs on gDNA isolated from NF54/NUP116-GFPDD clone C6 and Sanger sequencing again confirmed the simultaneous editing of both the *pfnup116* and *pfdhfr-ts* loci and the absence of wild-type parasites (Fig. S3).

Interestingly, and contrary to the previous IFA-based studies mentioned above (63), initial live cell fluorescence microscopy inspection of NF54/NUP116-GFPDD parasites cultured in the presence of Shield-1 revealed that PfNUP116-GFPDD localizes to a single spot at the nuclear periphery, but only in a small subset of cells, all of which were single-nucleated (Fig. S4). Withdrawal of the stabilizing ligand Shield-1 had no effect on parasite viability, and PfNUP116-GFPDD expression was still detectable in a minor fraction of parasites, albeit at markedly reduced levels (Fig. S4). Together, these observations suggested that (i) PfNUP116 may not be essential for asexual blood stage parasites; and (ii) PfNUP116 expression may be specifically induced in sexual ring stage parasites.

### PfNUP116 is not expressed in asexual parasites but specifically induced in sexual ring stage parasites

To test whether PfNUP116 is indeed specifically expressed in sexual ring stage parasites, we additionally tagged PfAP2-G with a triple hemagglutinin tag fused to mScarlet (HAmSc) in NF54/NUP116-GFPDD parasites. PfAP2-G is the master transcriptional regulator of sexual commitment and a specific marker for sexually committed schizonts, sexual ring stages (0–24 hpi), and early stage I gametocytes (24–35 hpi) (67–69). At the same time, we wished to demonstrate that CRISPR/Cas9^pyrR^-engineered parasites are amenable to iterative genetic engineering using h*dhfr* as a heterologous drug-selectable marker and the antifolate WR99210 as the selection drug. Although PYR and WR99210 both inhibit the parasite PfDHFR-TS enzyme, they bind distinct target sites (70), and PYR resistance-conferring mutations at N51, S108, and I164 are incompatible with WR99210 resistance (71). Hence, CRISPR/Cas9^pyrR^-engineered parasites are expected to retain sensitivity to WR99210. We, therefore, co-transfected NF54/NUP116-GFPDD parasites with the donor plasmid pD-*ap2g-hamSc* and the CRISPR/Cas9 suicide plasmid pH_gC-*ap2g-3*′ that carries a h*dhfr* resistance cassette (51, 72) (Fig. S5). Transfected parasites were selected on WR99210 (until day 6 post-transfection) and PYR (constantly), and a transgenic population carrying a successfully edited *pfap2-g* locus and free of unedited parasites was obtained approximately 3 weeks after transfection (Fig. S5).

**Fig 3:**
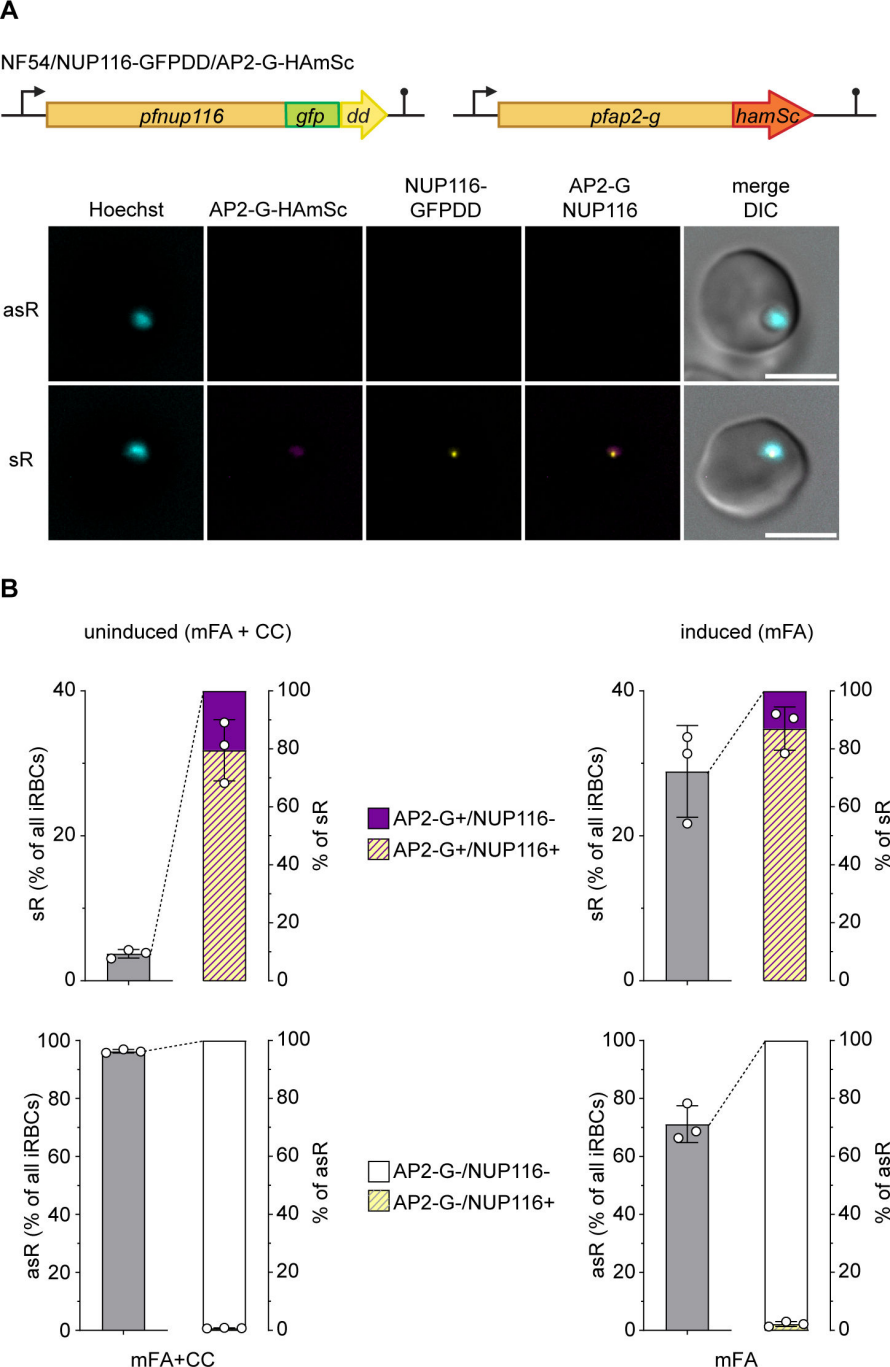
PfNUP116 is specifically expressed in sexual but not asexual ring stage parasites. (**A**) Top: Schematic illustration of the modified *pfnup116* and *pfap2-g* loci in NF54/NUP116-GFPDD/AP2-G-HAmSc parasites. Bottom: Representative live cell fluorescence images showing expression of PfNUP116-GFPDD specifically in PfAP2-G-HAmSc-positive sexual ring stages (sR) but not in asexual ring stages (asR). DNA was stained with Hoechst. DIC, differential interference contrast. Scale bar, 5 μm. (**B**) Quantification of PfAP2-G-HAmSc and PfNUP116-GFPDD co-expression in the late-ring stage progeny of NF54/NUP116-GFPDD/AP2-G-HAmSc parasites that were not induced (mFA + CC) or induced for sexual commitment (mFA). Values represent the mean of three independent biological replicates, and error bars represent the standard deviation. sR, sexual ring stages (PfAP2-G-HAmSc-positive); asR, asexual ring stages (PfAP2-G-HAmSc-negative); CC, choline chloride.

To test for co-expression of PfAP2-G and PfNUP116 at the single cell level, we performed live cell high content imaging of the late ring stage progeny of NF54/NUP116-GFPDD/AP2-G-HAmSc parasites exposed in the previous IDC to conditions that either suppress (mFA/2 mM choline chloride) or induce sexual commitment (mFA) (51). Based on the quantification of Hoechst- and PfAP2-G-HAmSc-derived signals, NF54/NUP116-GFPDD/AP2-G-HAmSc parasites cultured under non-inducing conditions displayed a sexual conversion rate (SCR) of 3.73% (±0.60% s.d.) (i.e., the proportion of sexual ring stage parasites among all ring stage parasites). Notably, most sexual ring stages (PfAP2-G-HAmSc-positive cells) also expressed PfNUP116-GFPDD (79.56%, ±10.56% s.d.) (Fig. 3A and B; Table S1). The pool of PfAP2-G-HAmSc-positive parasites that did not express PfNUP116-GFPDD (20.44%, ±10.56% s.d.) presumably represents younger sexual ring stages that did not yet express PfNUP116. In stark contrast, asexual parasites (PfAP2-G-HAmSc-negative cells) did not express PfNUP116-GFPDD (99.24%, ±0.11% s.d.) (Fig. 3A and B; Table S1). The negligible fraction of PfAP2-G-HAmSc-negative parasites that scored positive for PfNUP116-GFPDD expression (0.76%, ±0.11% s.d.) likely corresponds to early stage I gametocytes in which PfAP2-G expression already ceased. Highly congruent results were obtained with NF54/NUP116-GFPDD/AP2-G-HAmSc parasites cultured under sexual commitment-inducing conditions, which exhibited SCRs of 28.90% (±6.34% s.d.). Again, the vast majority of sexual ring stages (PfAP2-G-HAmSc-positive cells) also expressed PfNUP116-GFPDD (87.02%, ±7.46% s.d.) in contrast to only 2.24% (±0.87% s.d.) of asexual parasites (PfAP2-G-HAmSc-negative cells) (Fig. 3A and B; Table S1). Together, these results unequivocally demonstrate that PfNUP116 is specifically expressed in sexual but not asexual parasites.

### Localization dynamics of PfNUP116 during gametocyte development

**Fig 4:**
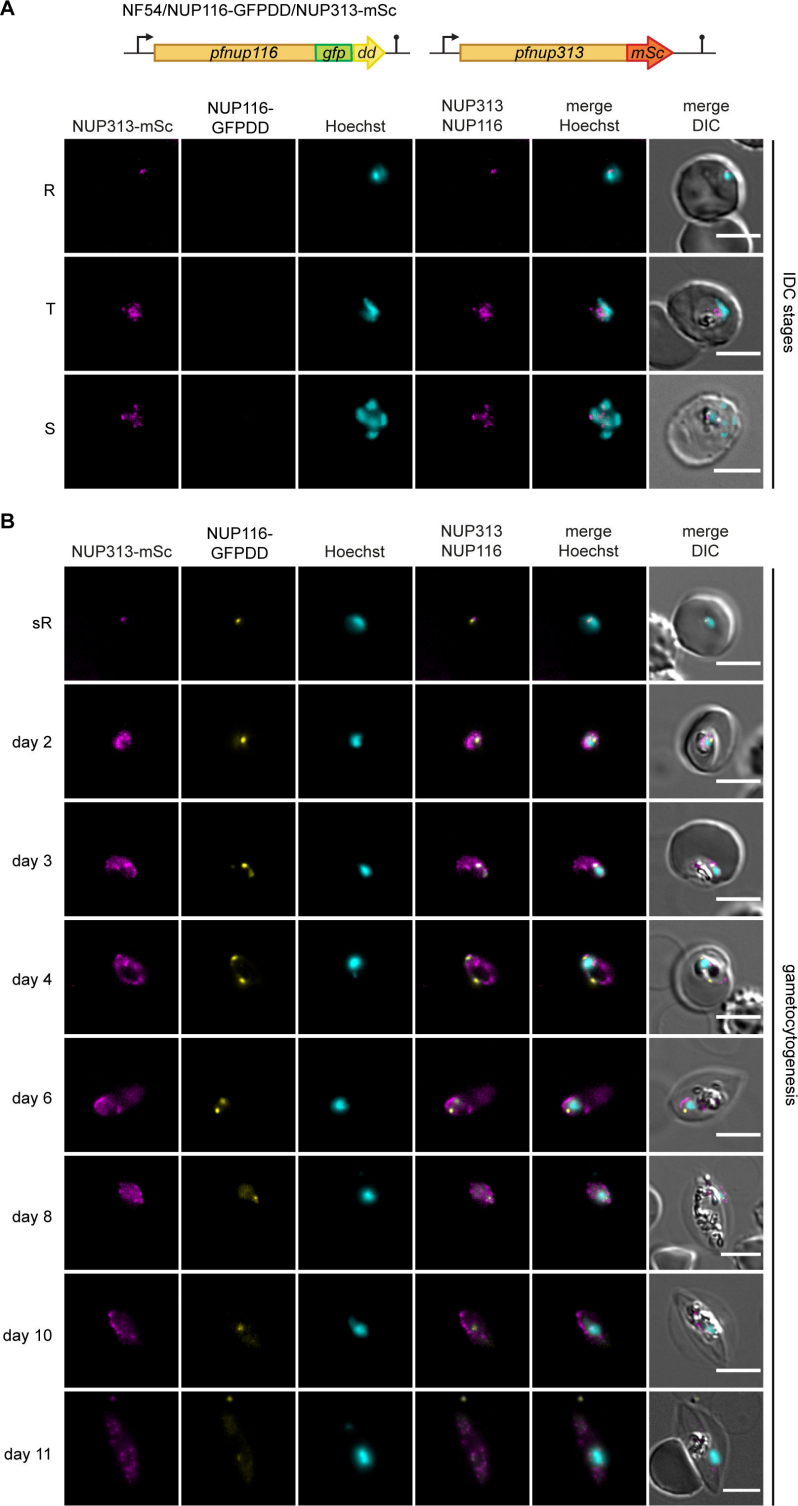
Expression and localization dynamics of PfNUP116 during gametocyte development. (**A**) Top: schematic illustration of the modified *pfnup116* and *pfnup313* loci in NF54/NUP116-GFPDD/NUP313-mSc parasites. Bottom: representative live cell fluorescence images showing expression of NUP313-mSc but not PfNUP116-GFPDD in asexual ring stages (R), trophozoites (T), and schizonts (S). (**B**) Representative live cell fluorescence images showing localization of PfNUP116-GFPDD in relation to the PfNUP313-mSc throughout gametocyte differentiation. DNA was stained with Hoechst. DIC, differential interference contrast; sR, sexual ring stage (day 1); days 2–11, days of gametocyte development. Scale bar, 5 μm.

To assess the expression and localization of PfNUP116 in greater detail, we again employed iterative gene editing, this time tagging PfNUP313 with mScarlet in NF54/NUP116-GFPDD parasites (Fig. S6). PfNUP313 is a nuclear pore protein for which we previously demonstrated expression in both asexual parasites and gametocytes (24). To this end, we co-transfected NF54/NUP116-GFPDD parasites with the CRISPR/Cas9 suicide plasmid pHF_gC-*nup313* and the donor plasmid pD-*nup313-mScarlet* (24). Transfected parasites were successfully selected on WR99210 and PYR, and a NF54/NUP116-GFPDD/NUP313-mSc clonal line carrying a correctly edited *pfnup313* locus free of unedited parasites was obtained after limiting dilution cloning (Fig. S6). In agreement with our earlier results, live cell fluorescence imaging of NF54/NUP116-GFPDD/NUP313-mSc parasites showed that in contrast to PfNUP313-mSc, PfNUP116-GFPDD was not expressed at detectable levels in asexual blood stage parasites (Fig. 4A). In late sexual ring stages, PfNUP116-GFPDD and PfNUP313-mSc co-localized at a single perinuclear site (Fig. 4B). Interestingly, PfNUP116-GFPDD remained confined to this site in stage I gametocytes (day 2 of gametocyte development), whereas PfNUP313-mSc began to spread across the NE. In stage II and III gametocytes (days 3–7), as the gametocyte cell body and nucleus elongate, we frequently observed a second perinuclear PfNUP116-GFPDD dot (Fig. 4B). During later gametocyte maturation, the PfNUP116-GFPDD signal gradually weakened, adopting a more dispersed pattern and was nearly undetectable in mature stage V gametocytes. Furthermore, we observed that NF54/NUP116-GFPDD/NUP313-mSc gametocytes cultured in the absence of Shield-1 progressed until stage V without any developmental delay despite a ~6 fold reduction in NUP116-GFPDD expression levels (Fig. S7), suggesting that PfNUP116 is not essential for gametocytogenesis. However, alternative conditional knockdown or knockout approaches will be required to firmly establish whether PfNUP116 is indeed dispensable for gametocyte development.

In summary, these data establish PfNUP116 as a distinct nuclear periphery marker in early gametocytes, revealing dynamic compositional changes of this nuclear compartment during sexual differentiation and warranting further investigations into the function of this gametocyte-specific factor.

## DISCUSSION

In the present study, we introduce CRISPR/Cas9^pyrR^ as a CRISPR/Cas9-based gene editing method for *P. falciparum*, which combines concurrent editing of a gene of interest with the insertion of resistance-conferring mutations into the *pfdhfr-ts* gene, enabling selection of transgenic parasites using the antimalarial drug PYR. This approach eliminates the need for a heterologous drug-selectable marker, offering a gene editing strategy that can be used in conjunction with subsequent CRISPR/Cas9 methodologies relying on established heterologous drug resistance markers for iterative gene editing. Notably, by utilizing two separate plasmids to deliver the SpCas9 expression cassette (plasmid 1) and the *dhfr^pyrR^* donor sequence along with a *pfdhfr-ts*-targeting sgRNA (plasmid 2), CRISPR/Cas9^pyrR^ ensures that only parasites that acquired both plasmids and underwent immediate editing of the *pfdhfr-ts* locus can survive under PYR selection pressure. This dependence on both plasmids explains why efficient co-editing of both loci was observed in both CRISPR/Cas9^pyrR^-engineered lines reported here, even though there is no direct selection pressure on parasites that modified the gene of interest using the corresponding donor template and sgRNA provided with plasmid 1. The fact that the initially selected PYR-resistant populations consisted of a mixture of parasites carrying edited or unedited loci (Fig. S2 and S3) is not a limitation specific to the CRISPR/Cas9^pyrR^ protocol, but is commonly observed with CRISPR/Cas9 gene editing approaches presently applied in *P. falciparum*, as reported here (Fig. S6) and elsewhere (12, 19, 21, 22) and likely primarily dependent on sgRNA efficiency (73). Similarly, integration of one or several copies of the entire donor plasmid (Figs. S3, S5 and S6), rather than just the donor repair module, is frequently observed after transfection of circular plasmids targeting non-essential genes or designed for 3′ end tagging (7, 22, 24, 74, 75). The underlying reasons for these events are not entirely clear, but they probably occur when parasites use episomal donor plasmid concatamers, which rapidly form in *P. falciparum* due to a rolling circle-like mode of plasmid replication (76), as the template for HDR.

In practice, CRISPR/Cas9^pyrR^ simply requires the cloning of the sgRNA and donor template targeting the gene of interest into plasmid 1 (p_gCD_*goi*), while plasmid 2 (p_gD-*dhfr*^pyrR^), which carries the sgRNA targeting *pfdhfr-ts* and the *dhfr^pyrR^* donor template, is universally utilized in all transfections. To illustrate this principle, we applied CRISPR/Cas9^pyrR^ to engineer transgenic parasites expressing either PfNUP116-GFPDD or PfGEX1-GFP. We furthermore demonstrated the feasibility of subjecting CRISPR/Cas9^pyrR^-derived transgenic parasites to a second round of genetic engineering using the heterologous drug resistance marker hDHFR combined with selection on WR99210. Here, we advise on the combined use of WR99210 and PYR for the selection of transgenic parasites; even though to our knowledge, WR99210-resistant parasites have never been reported, the screening of an *Escherichia coli* library expressing random PfDHFR-TS mutants identified variants resistant to WR99210 but not against the combination of WR99210 and PYR (71).

Analysis of the NF54/GEX1-GFP line showed that PfGEX1-GFP is not expressed in asexual blood stage parasites but specifically in late-stage gametocytes where it localizes to the NE. This observation aligns with findings on the PbGEX1 ortholog in *P. berghei* that similarly localizes to the NE in gametocytes and persists through to the ookinete stage (46, 47). Notably, *pbgex1* knock-out parasites show defective oocyst formation and fail to produce sporozoites (46), indicating a critical role in malaria transmission. Whether PfGEX1 serves a similar function in sexual development in *P. falciparum* remains to be determined. Interestingly, stage-specific expression of NE proteins in malaria parasites has recently also been described for SUN-domain proteins, which constitute conserved elements of the linker of nucleoskeleton and cytoskeleton (LINC) complex that spans the inner and outer nuclear membranes in other eukaryotes (77, 78). In *P. berghei*, a putative PbSUN1–PbALLAN complex was recently proposed to form part of a divergent LINC-like complex in gametocytes and was shown to be required for MTOC organization and mitosis during male gametogenesis (79, 80). In *P. falciparum*, two SUN-domain proteins, PfSUN1 and PfSUN2, were recently demonstrated to localize to the NE in asexual blood stage parasites and to be essential for schizogony (81), but their roles during gametocytogenesis remain to be investigated.

Our analysis of the double-transgenic lines NF54/NUP116-GFPDD/AP2-G-HAmSc and NF54/NUP116-GFPDD/NUP313-mSc challenges previous reports by revealing that the putative nucleoporin PfNUP116 is not expressed in asexual blood stage parasites but exclusively in gametocytes. Based on live cell fluorescence microscopy, NUP116-GFPDD expression becomes detectable in late sexual ring stages, where it localizes to a distinct perinuclear site in close proximity to the nuclear pore protein PfNUP313. From stage I onwards, this spatial association progressively diminishes as PfNUP313 redistributes across the NE. By day 3 of gametocyte development, a second PfNUP116 focus is often detected, located away from the initial perinuclear region. These observations suggest that PfNUP116 may not be a canonical component of NPCs but could instead be associated with a distinct subset of nuclear pores or another nuclear structure during early gametocytogenesis.

In summary, we show that the CRISPR/Cas9^pyrR^ system developed here provides a suitable and equally effective alternative approach for CRISPR/Cas9-based gene editing in *P. falciparum* compared to presently applied methods. Note, however, that CRISPR/Cas9^pyrR^ can only be used for PYR-sensitive parasite strains, such as NF54 and 3D7, but not for other strains like 7G8, Dd2/W2-Mef, or V1/S that already carry PYR resistance-conferring mutations in their endogenous *pfdhfr-ts* gene (30, 31, 40, 41). Our preliminary analysis of two CRISPR/Cas9^pyrR^-engineered NF54 parasite lines identified PfNUP116 and PfGEX1 as novel nuclear markers for early- and late-stage *P. falciparum* gametocytes, respectively. Because CRISPR/Cas9^pyrR^ does not rely on the use of heterologous drug resistance markers, we believe it is particularly suited for research requiring iterative gene editing. Furthermore, we envisage that a conceptually similar gene editing strategy may be applicable to other endogenous drug resistance alleles known to confer high-level resistance to other antimalarial drugs, such as those encoding the chloroquine resistance transporter PfCRT (82, 83) or hydroxymethyldihydropterin pyrophosphokinase-dihydropteroate synthetase (PPPK-DHPS) (sulphadoxine resistance) (84).

## MATERIALS AND METHODS

### Parasite culture

*P. falciparum* parasites were cultured using human RBCs of blood group AB+ (Blutspende SRK Zürich, Switzerland) at a hematocrit of 5% in RPMI 1640 medium (10.44 g/L) supplemented with 25 mM HEPES, 370 μM hypoxanthine, 24 mM sodium bicarbonate, 100 μg/mL neomycin, and 0.5% Albumax II (Gibco #11021-037). In addition, 2 mM choline chloride was added to the culture medium to suppress sexual commitment (51). For NF54/NUP116-GFPDD parasites, 700 nM Shield-1 was routinely added to the growth medium to prevent degradation of the FKBP/DD-tagged fusion protein. Repeated sorbitol treatments were used for intra-erythrocytic growth synchronizations (85). Parasite cultures were maintained at 37°C in airtight chambers in an atmosphere of 3% O_2_, 4% CO_2_, and 93% N_2_.

### Transfection constructs

The p_gD-*dhfr*^pyrR^ plasmid was obtained by replacing the SpCas9 expression cassette in plasmid p_gC(20) with a previously assembled 1,495 bp donor sequence (positions ‒445 to +1,050 with regard to the ATG start codon) encoding four amino acid changes that confer high-level resistance to PYR (N51I, C59R, S108N, I164L) (*dhfr^pyrR^*). The *dhfr^pyrR^* donor sequence was generated and subcloned into a pUC19 vector by Gibson assembly (86, 87) of five PCR fragments. Fragments 1–4 cover bp ‒445 to +162 (primers AO_DHFR-TS_5′_F and DHFR-TS_5′_R), bp +139 to +328 (primers DHFR-TS_rec_F1 and DHFR-TS_rec_R1), bp +300 to +502 (primers DHFR-TS_rec_F2 and DHFR-TS_rec_R2), and bp +481 to +1050 (primers DHFR-TS_3′_F and AO_DHFR-TS_3′_R) of the *pfdhfr-ts* gene, respectively. Fragments 2 and 3 were amplified from a synthetic *pfdhfr* sequence (Genscript) that encodes the N51I, C59R, S108N, and I164L mutations and is re-codonized to avoid cross-over events in the region spanning these four mutations (bp +150 to +492). Fragments 1 and 4 were amplified from 3D7 gDNA with mutated/re-codonized residues contained in the DHFR-TS_5′_R and DHFR-TS_3′_F primers, respectively. Fragment 5 represents the plasmid backbone amplified from pUC19 amplified with primers PCRA_F and PCRA_R(20). This intermediate vector (pAO_DHFR_TS_HBox) was then used as a template for PCR amplification of the entire *dhfr^pyrR^* sequence assembly using primers BspHI_F and HindIII_R. To generate the final p_gD-*dhfr*^pyrR^ transfection vector, two subsequent T4 DNA ligase reactions were performed. First, the SpCas9 expression cassette contained in the CRISPR/Cas9 vector p_gC has been excised with NcoI and HindIII and replaced with the BspHI/HindIII-digested *dhfr^pyrR^* donor sequence. Second, annealed complementary oligonucleotides encoding a sgRNA targeting the *pfdhfr-ts* coding sequence (bp 305–324) were inserted into the BsaI site of the sgRNA expression cassette.

Prior to generating p_gCD plasmids, plasmid pD-*gfpdd*-BsaI was created to mutate the BsaI site within the coding region of *gfp* to be used as a template for *gfp* and *gfpdd* sequences (since the BsaI site in p_gCD plasmids is used to insert the sgRNA). The *gfpdd* sequence was amplified from plasmid pFdon_*map-2gfpdd* (88) in two fragments, introducing two synonymous mutations in *gfp* (A645G and C648T) using primer combinations gfpdd_F1 and gfpdd_R1, and gfpdd_F2 and gfpdd_R2, respectively. These two fragments were inserted into the plasmid backbone of pUC19 (amplified with primers PCRA_F and PCRA_R) in a three-fragment Gibson assembly reaction.

Plasmid p_gCD-*gex1-gfp* has been obtained by digesting plasmid p_gC with HindIII and BamHI and inserting a donor sequence module consisting of a 5′ homology box (HB) representing the last 997 bp of the *pfgex1* coding sequence (bp +2524–3520; amplified from NF54 gDNA using primers gex1_5′_F and gex1_5′_R), a sequence encoding GFP (amplified from plasmid pD-*gfpdd*-BsaI using primers gex1_gfp_F and gex1_gfp_R), a sequence encoding the *P. falciparum hrp2* terminator (amplified from plasmid p_gCH-*pfap2-hc-KO* (75) using primers gex1_hrp2_F and gex1_hrp2_R), and a 3′ HB representing bp 290–1445 downstream of the *gex1* STOP codon (amplified from NF54 gDNA using primers gex1_3′_F and gex1_3′_R) using a five-fragment Gibson assembly reaction. Subsequently, annealed complementary oligonucleotides encoding a sgRNA targeting the downstream region of the *pfgex1* coding sequence (bp +199–218) were inserted into the BsaI site of the sgRNA expression cassette using a T4 DNA ligase reaction.

Plasmid p_gCD-*nup116-gfpdd* was obtained by digesting plasmid p_gC with HindIII and BamHI and inserting a donor sequence module consisting of three fragments. Fragment 1 consists of a 5′ HB that encodes the last 767 bp of the *pfnup116* coding sequence (bp +2993–3759) and was amplified from a synthetic *pfnup116* sequence cloned into pUC57 (GenScript) (using primers nup116_5′_rec_F + nup116_5′_rec_R), where bp +3414–3759 have been re-codonized to mutate the sgRNA binding site. Fragment 2 encodes GFPDD (amplified from plasmid pD-*gfpdd*-BsaI using primers nup116_gfpdd_F and nup116_gfpdd_R), and fragment 3 encodes a 3′ HB representing the 722 bp downstream of the *pfnup116* STOP codon (amplified from NF54 gDNA using primers nup116_3′_F and nup116_3′_R). Fragments 1–3 were assembled with the digested p_gC plasmid in a four-fragment Gibson assembly reaction. Subsequently, annealed complementary oligonucleotides encoding a sgRNA targeting the *pfnup116* coding sequence (sgRNA_nup116_1: bp +3412–3431) were inserted into the BsaI site of the sgRNA expression cassette using a T4 DNA ligase reaction.

The plasmid pHF_gC-*nup313* was obtained by inserting annealed complementary oligonucleotides encoding a sgRNA targeting the region downstream of the *pfnup313* STOP codon (bp +91–110) (24) into the BsaI site of the sgRNA expression cassette in pHF_gC (20, 24) using a T4 DNA ligase reaction. The plasmid pD-*ap2-g-hamSc* was generated by digesting plasmid pD_*ap2g-m*Scarlet (72) with BamHI and inserting a single fragment encoding a triple hemagglutinin (3xHA) tag (amplified from plasmid pBcam-3xHA [89] using primers ha_F and ha_R) in a Gibson assembly reaction.

All primers used for plasmid cloning are listed in Table S2.

### Transfection and selection of CRISPR/Cas9-edited transgenic cell lines

For PYR-dependent selection of CRISPR/Cas9-edited parasites (CRISPR/Cas9^pyrR^), *P. falciparum* NF54 ring stage parasites were co-transfected by electroporation with p_gD-*dhfr*^pyrR^ and either p_gCD-*gex1-gfp* (NF54/GEX1-GFP) or p_gCD-*nup116-gfpdd* (NF54/NUP116-GFPDD) (50 μg plasmid DNA each). On the day after transfection, the culture medium was exchanged and 500 nM PYR added for three subsequent days, with daily medium replacement. From day 4 onwards, parasites were cultured constantly in the presence of 200 nM PYR. For parasites transfected with p_gCD-*nup116-gfpdd*, 700 nM Shield-1 (+Shield-1) was constantly added to the culture medium to stabilize PfNUP116-GFPDD expression. Once stably growing transgenic populations were obtained (3–4 weeks after transfection), correct editing of the *pfdhfr-ts* and *pfgex1*/*pfnup116* loci was confirmed by PCRs on gDNA. Clonal NF54/GEX1-GFP and NF54/NUP116-GFPDD parasite populations were obtained by limiting dilution cloning as described (90) and correct editing of the *pfdhfr-ts* and *pfgex1*/*pfnup116* genes confirmed by PCR on gDNA. The presence of the PYR resistance-conferring mutations in the edited *pfdhfr-ts^pyrR^* gene was confirmed by Sanger sequencing. Sequence data analysis and visualization were performed using SnapGene software 5.1.2. (Insightful Science, San Diego, CA, USA).

NF54/NUP116-GFPDD parasites (200 nM PYR, 700 nM Shield-1) were co-transfected as explained above with the CRISPR/Cas9 suicide plasmid pHF_gC-*nup313* and donor plasmid pD-*nup313-mScarlet* (24) to obtain the NF54/NUP116-GFPDD/NUP313-mSc line. Likewise, the NF54/NUP116-GFPDD/AP2-G-HAmSc line was obtained by co-transfection of the CRISPR/Cas9 suicide plasmid pH_gC-*ap2g-3*′ (51) and donor plasmid pD-*ap2-g-hamSc*. On the day after transfection, the culture medium was exchanged and 4 nM WR99210 added for six subsequent days, with daily medium replacement. From day 7 onwards, parasites were cultured in the absence of WR99210 and constant presence of 200 nM PYR. Once stably growing transgenic populations were obtained (3–4 weeks after transfection), correct editing of the *pfnup313* or *pfap2-g* genes was confirmed by PCRs on genomic DNA (gDNA). Clonal NF54/NUP116-GFPDD/NUP313-mSc parasite populations were obtained by limiting dilution cloning and correct editing of the *pfnup313* gene confirmed by PCR on gDNA. All primers used for PCR on gDNA are listed in Table S2.

### Induction of sexual commitment and gametocyte maturation

Sexual commitment was induced by growing synchronous late ring stage parasites (16–24 hpi) for one replication cycle in minimal fatty acid (mFA) medium containing 0.39% fatty acid-free BSA (Sigma #A6003)/30 μM oleic acid (Sigma #O1008)/30 μM palmitic acid (Sigma #P0500) instead of 0.5% Albumax II (51). Parasites cultured in parallel in mFA medium supplemented with 2 mM choline chloride to suppress sexual commitment were used as a control. The ring stage progeny (asexual and sexual ring stages; day 1 of gametocyte differentiation) was cultured in normal culture medium for subsequent high content microscopy experiments or in culture medium containing 10% heat-inactivated AB+ human serum (Blood Donation Center, Basel, Switzerland) instead of 0.5% Albumax II for the preparation of Hemacolor-stained thin blood smears and live cell fluorescence imaging throughout gametocytogenesis. During the first 6 days of gametocyte maturation, the culture medium was supplemented with 50 mM N-acetylglucosamine (GlcNAc) to eliminate asexual parasites (52). Thereafter, gametocytes were cultured in the absence of GlcNAc until the end of gametocyte maturation (day 12; stage V gametocytes).

### Live cell fluorescence microscopy

Culture aliquots (0.5–1 mL) were centrifuged at 600 × *g* and RBC pellets resuspended in 0.5 mL medium containing 5 µg/mL Hoechst 33342 (Thermo Fisher Scientific #H3570) to stain the parasite DNA (20 min at 37°C). Stained RBCs were washed once in PBS and mounted on microscopy slides using Vectashield (Vector Laboratories). Images were acquired on either a Leica DM5000 B fluorescence microscope (63× objective) or a Leica Thunder 3D assay fluorescence microscope (63× objective) equipped with Leica K5 sCMOS cameras. Both microscopes used the Leica application suite X software (LAS X version 3.7.5.24914). Image processing was performed with Fiji (ImageJ version 1.52n). Within each experiment, all images were acquired and processed with identical settings.

### High-content microscopy and image analysis

The proportion of PfNUP116-GFPDD-positive, PfAP2-G-HAmScarlet-positive, double-positive, and double-negative NF54/NUP116-GFPDD/AP2-G-HAmSc parasites at 18–26 hpi in the generation following the induction of sexual commitment was quantified using high-content microscopy and automated image analysis as described previously (72). Parasite suspensions were stained with 5 µg/ml Hoechst 33342 (Thermo Fisher Scientific #H3570) for 20 min. The RBCs were pelleted, washed once with PBS, and resuspended in PBS at a hematocrit of 0.05%. Subsequently, 200 μL aliquots were transferred to the wells of a clear-bottom 96-well plate suitable for high-content microscopy (Greiner Bio-One #655090). Thirty-six sites per well were acquired using the ImageXpress Micro XLS wide-field, high-content screening system (Molecular Devices) with a 40× Plan-Apochromat objective (Molecular Devices #1-6300-0297) in the GFP (exciter [Ex] center/bandwidth: 472/30 nm, emitter [Em] center/bandwidth: 520/35 nm, exposure time: 400 ms), TRITC (Ex: 543/22 nm, Em: 593/40 nm, exp: 80 ms), and DAPI (Ex: 377/50 nm, Em: 447/60 nm, exp: 80 ms) channels. Image analysis was performed using the MetaXpress software (Molecular Devices version 6.5.4.532). Infected RBCs (iRBCs) were identified based on their Hoechst intensity (DAPI channel: intensity above local background > 2,000, size = 0.9–3.2 μm) and further categorized into either one of four groups based on their fluorophore intensities: (i) GFP-positive (GFP channel: intensity above local background > 300, size = 0.9–3.2 μm); (ii) mScarlet-positive (TRITC channel: intensity above local background > 400, size = 0.9–3.2 μm); (iii) GFP/mScarlet double-positive; or (iv) GFP/mScarlet double-negative. The total number of iRBCs captured per well and the number of cells quantified per group are listed in Table S1. Experiments were performed in three independent biological replicates.

### Flow cytometry

NF54/NUP116-GFPDD/NUP313-mSc parasites were split at the early ring stage, and Shield-1 was either maintained or removed from the culture medium. Both cultures were then induced for sexual commitment as described above, and their ring stage progeny were cultured in the presence of 50 mM GlcNAc for one additional day. On day 2 after invasion (mixture of stage I gametocytes and asexual parasites), culture aliquots were stained using 5 µg/mL Hoechst 33342 (Thermo Fisher Scientific #H3570) for 20 min at 37°C protected from light, and then washed in PBS. Using a MACS Quant Analyzer 10, 10,000 events per condition were measured and analyzed for Hoechst and GFP fluorescence using the FlowJo_v10.6.1 software. The gating strategy is shown in Fig. S7.

## Data Availability

All data linked to this study are provided in the main article and supplemental information.

## SUPPLEMENTAL MATERIAL

Supplemental figures and tables msphere.00884-25-s0001.pdf Figures S1–S7; Tables S1 and S2.

ASM does not own the copyrights to Supplemental Material that may be linked to, or accessed through, an article. The authors have granted ASM a non-exclusive, world-wide license to publish the Supplemental Material files. Please contact the corresponding author directly for reuse.
