# Supplementary material for: A heterologous marker-free selection approach for CRISPR/Cas9-based gene editing in the malaria parasite Plasmodium falciparum
Source: mSphere. 2026 Mar 26;11(4):e00884-25. doi: 10.1128/msphere.00884-25 (PMC13123713; doi:10.1128/msphere.00884-25)
Supplement: Supplemental figures and tables — Figures S1–S7; Tables S1 and S2. [file msphere.00884-25-s0001.pdf]

SUPPLEMENTARY INFORMATION

A heterologous marker-free selection approach for CRISPR/Cas9-based gene editing in the malaria parasite *Plasmodium falciparum*

Eilidh Carrington[1,2], Daniel Ballmer[1,2], Igor Niederwieser[1,2], Basil T. Thommen[1,2,#], Nicolas M. B. Brancucci[1,2], Till S. Voss[1,2,*]

[1]Department of Medical Parasitology and Infection Biology, Swiss Tropical and Public Health Institute, 4123 Allschwil, Switzerland.
[2]University of Basel, 4001 Basel, Switzerland.

[#]Current address: Department of Immunology and Infectious Diseases, Harvard T. H. Chan School of Public Health, Boston, MA, 02115, USA.

[*]Corresponding author: till.voss@swisstph.ch.

This Supplementary Information file includes:

- Figures S1-S7
- Tables S1-S2

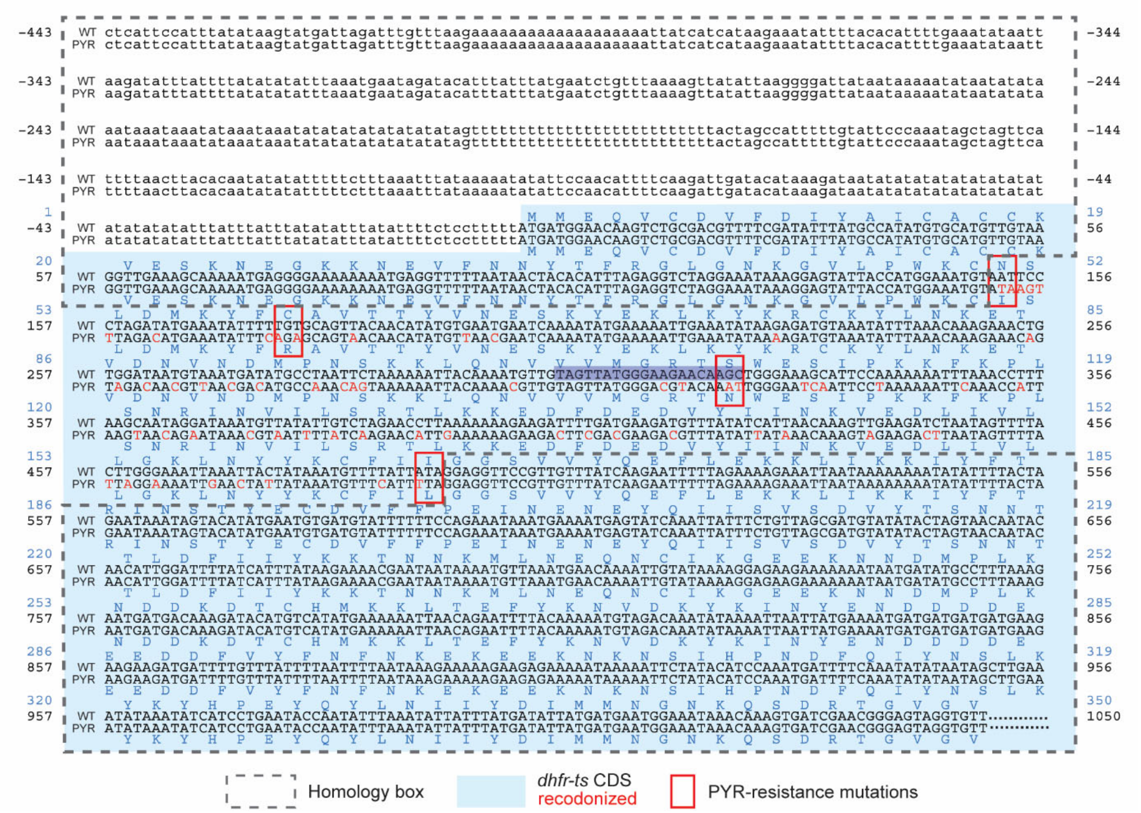

Figure S1. Pairwise nucleotide sequence alignment of parts of the upstream and coding sequence of the wild-type *pfdhfr-ts* gene (WT) and the repair sequence provided on the p_gD-*dhfr*^{PyrR} donor plasmid. The 5' and 3' homology boxes are highlighted by dashed grey boxes. The 5' end of the *pfdhfr-ts* coding sequence is shaded light blue. Silent mutations in the recodonised *dhfr*^{pyrR} sequence are highlighted in red letters. Non-synonymous mutations resulting in the N51I, C59R, S108N, I164L PYR resistance-conferring mutations are highlighted by red boxes. The sgRNA target site is shaded dark blue. Amino acids are represented by the one letter code and shown in blue letters above and below the nucleotide sequence alignment. Numbers in black/blue letters at the left and right sides of the alignment refer to the nucleotide/amino acid positions relative to the Start codon/methionine, respectively.

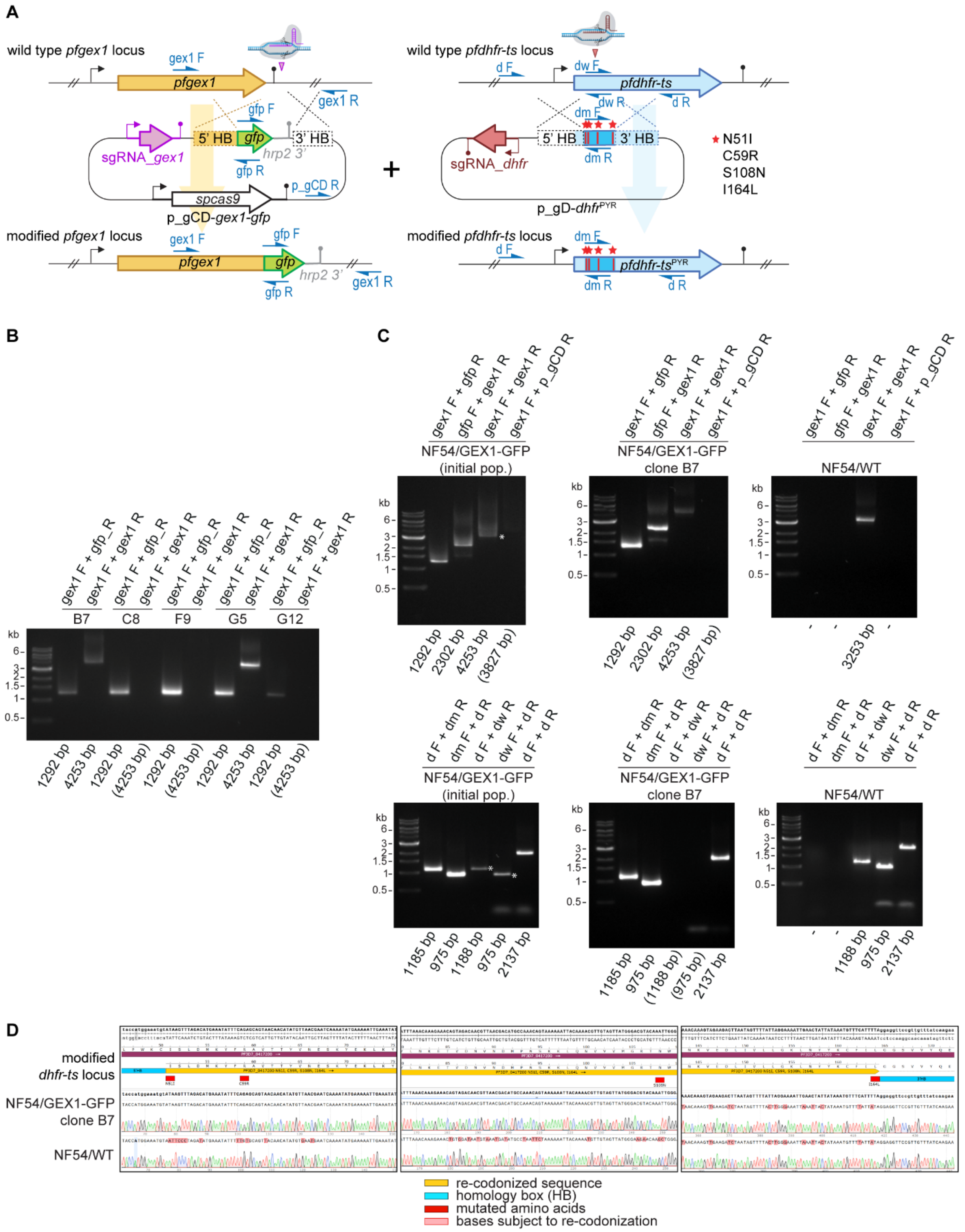

Figure S2. Cloning strategy and validation of the NF54/GEX1-GFP cell line. (A) Schematic illustration of the wild-type (top) and modified (bottom) *pfgex1* and *pfdhfr-ts* loci, along with the two CRISPR/Cas9^{pyrR} plasmids (p_gCD_*gex1-gfp* and p_gD-*dhfr*^{pyrR}) used to engineer the NF54/GEX1-GFP parasite line. Names and relative positions of primers used for diagnostic PCRs are indicated by horizontal arrows. HB, homology box; *hrp2 3'*, histidine-rich protein 2 terminator. **(B)** PCRs performed on gDNA extracted from five NF54/GEX1-GFP clonal populations for the *pfgex1* locus. Names of

primer pairs used for diagnostic PCRs are indicated on top. Expected DNA fragment sizes are given below the gel images, with non-amplified fragments indicated in brackets. The absence of the 4,253 bp band in the successfully edited clones C8, F9 and G12 is suggestive of donor plasmid integration. kb, kilo base; bp, base pairs. **(C)** PCRs performed on gDNA extracted from the initially selected transgenic NF54/GEX1-GFP population (initial pop.) (left), NF54/GEX1-GFP clone B7 (middle) and NF54 wild type control parasites (right) for the *pfgex1* locus (top panel) and the *pfdhfr-ts* locus (bottom panel). Names of primer pairs used for diagnostic PCRs are indicated on top. Expected DNA fragment sizes are given below the gel images, with non-amplified fragments indicated in brackets. Asterisks mark the presence of unedited target loci in the initially selected transgenic population. kb, kilo base; bp, base pairs; WT, wild type. **(D)** Chromatogram from Sanger sequencing of the PCR products obtained with primers d_F/d_R from NF54/GEX1-GFP clone B7 and NF54/WT gDNA demonstrating successful editing of the *pfdhfr-ts* locus. Note that only three of the four PYR resistance-conferring point mutations have been introduced at the modified $pfdhfr\text{-}ts^{PYR}$ locus (N51I, C59R, S108N) due to a 3' crossover event upstream of the I164L codon.

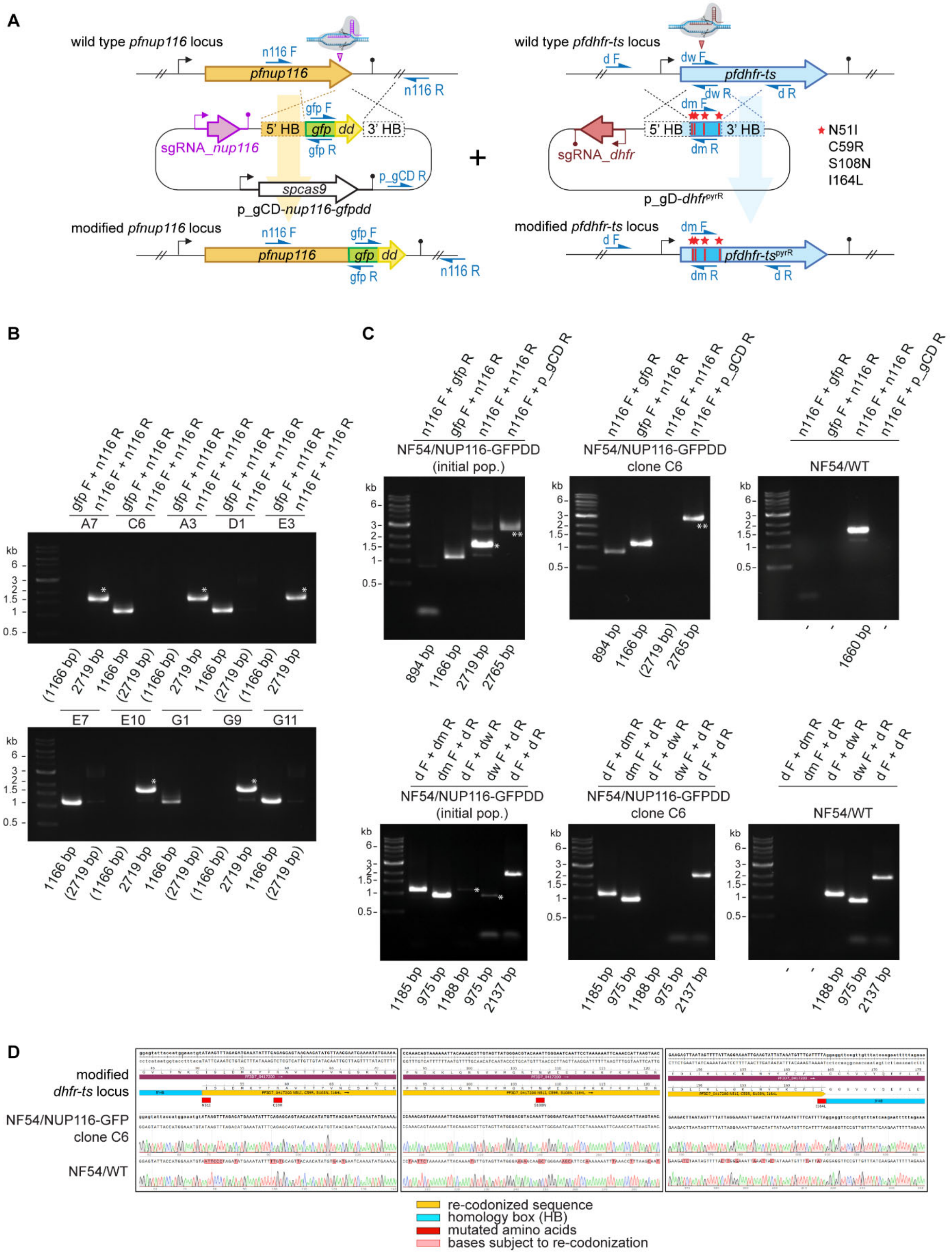

Figure S3. Cloning strategy and validation of the NF54/NUP116-GFPDD cell line. (A) Schematic illustration of the wild-type (top) and modified (bottom) *pfnup116* and *pfdhfr-ts* loci, along with the two CRISPR/Cas9^{pyrR} plasmids (p_gCD_*nup116-gfp* and p_gD-*dhfr*^{pyrR}) used to engineer the NF54/NUP116-GFPDD parasite line. Names and relative positions of primers used for diagnostic PCRs

are indicated by horizontal arrows. HB, homology box. **(B)** PCRs performed on gDNA extracted from ten NF54/NUP116-GFPDD clonal populations for the *pfnup116* locus. Names of primer pairs used for diagnostic PCRs are indicated on top. Expected DNA fragment sizes are given below the gel images, with non-amplified fragments indicated in brackets. The absence of the 2,719 bp band in all five successfully edited clones (C6, D1, E7, G1 and G11) is suggestive of donor plasmid integration. Single asterisks mark the presence of unedited target loci in clones A7, A3, E3, E10 and G9. kb, kilo base; bp, base pairs. **(C)** PCRs performed on genomic DNA extracted from the initially selected transgenic NF54/NUP116-GFPDD population (initial pop.) (left), NF54/NUP116-GFPDD clone C6 (middle) and NF54 wild type control parasites (right) for the *pfnup116* locus (top panel) and the *pfdhfr-ts* locus (bottom panel). Names of primer pairs used for diagnostic PCRs are indicated on top. Expected DNA fragment sizes are given below the gel images, with non-amplified fragments indicated in brackets. Single asterisks mark the presence of unedited target loci in the initially selected transgenic population. Double asterisks indicate p_gCD_*nup116-gfp* donor plasmid integration downstream of the tagged *pfnup116* gene. kb, kilo base; bp, base pairs; WT, wild type. **(D)** Chromatogram from Sanger sequencing of the PCR products obtained with primers d_F/d_R from NF54/NUP116-GFPDD clone C6 and NF54/WT gDNA showing successful editing of the *dhfr-ts* locus.

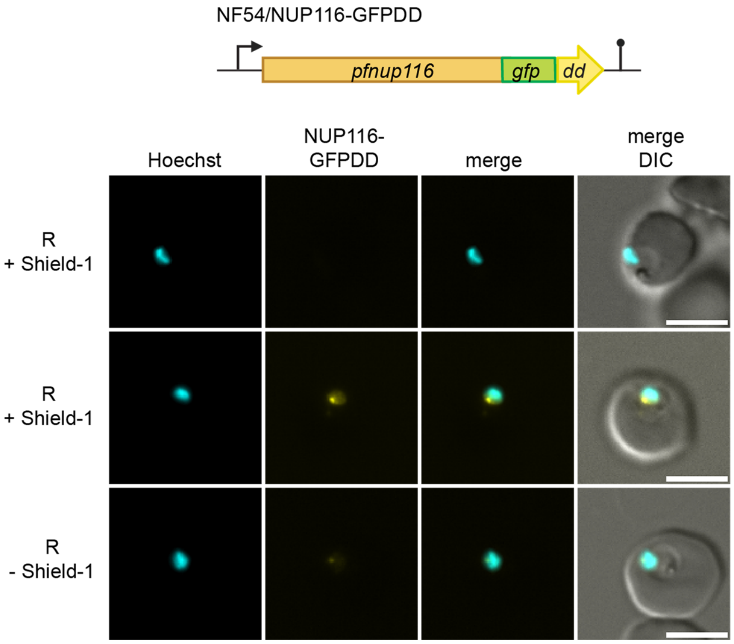

Figure S4. PfNUP116 is only expressed in a subset of single-nucleated parasites and localizes to a single perinuclear spot. Representative live cell fluorescence images showing PfNUP116-GFPDD expression in NF54/NUP116-GFPDD ring stage parasites cultured in the presence or absence of Shield-1**.** DNA was stained with Hoechst. DIC, differential interference contrast; R, ring stage. Scale bar, 5 µm.

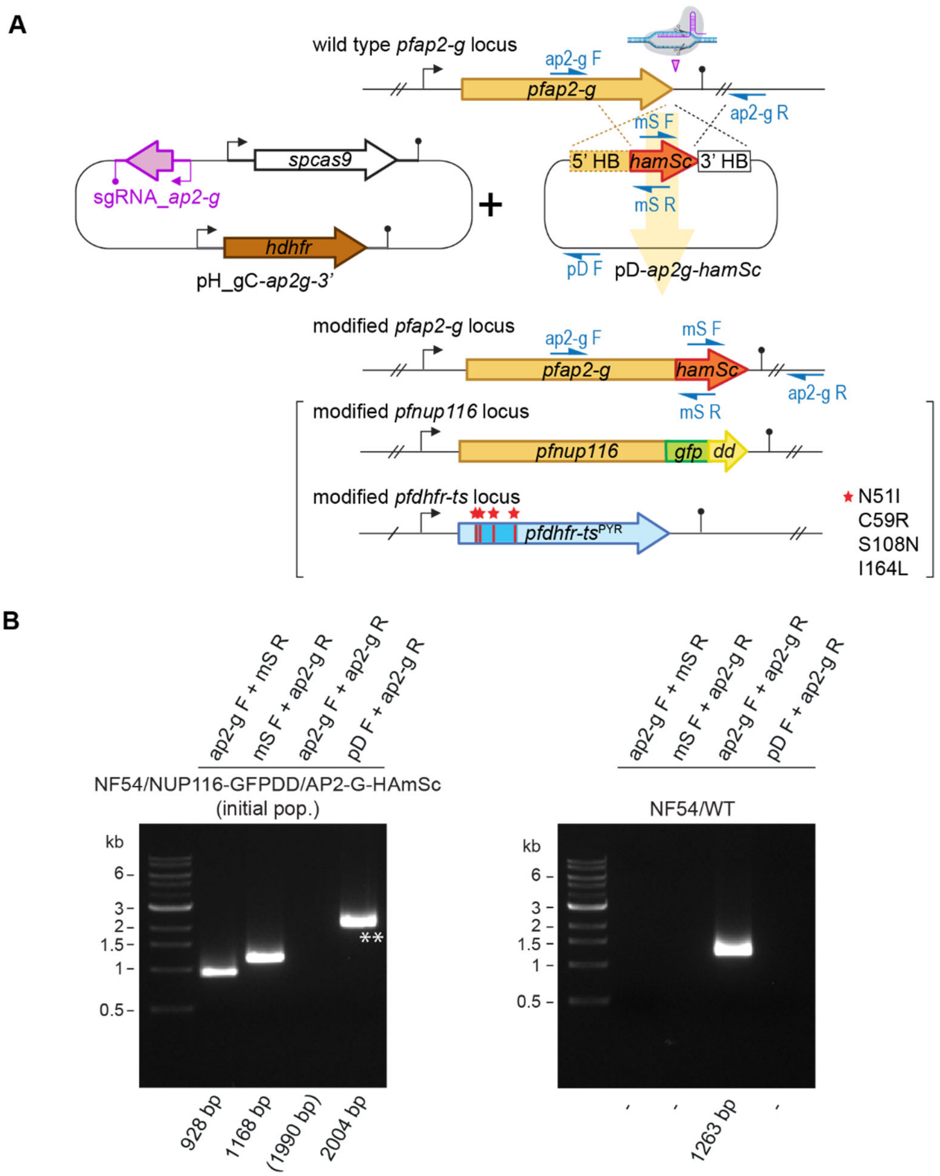

Figure S5. Cloning strategy and validation of the NF54/NUP116-GFPDD/AP2-G-HAmSc double transgenic cell line. (A) Schematic illustration of the wild-type and modified *pfap2-g* loci, alongside the plasmids (pH_gC-*ap2g-3'* and pD-*ap2g-hamSc*) used for iterative editing of the NF54/NUP116-GFPDD parasite line. Selection of gene-edited parasites was facilitated using the h*dhfr* drug resistance marker combined with WR99210-based selection. The previously modified *pfnup116-gfpdd* and *pfdhfr-ts*^{pyrR} loci are shown below for reference. Names and relative positions of primers used for diagnostic PCRs are indicated by horizontal arrows. HB, homology box. **(B)** PCRs performed on the *pfap2-g* locus on genomic DNA extracted from the initially selected transgenic NF54/NUP116-GFPDD/AP2-G-HAmSc population (initial pop.) (left) and NF54 wild type control parasites (right). Names of primer pairs used for diagnostic PCRs are indicated on top. Expected DNA fragment sizes are given below gel images, with non-amplified fragments indicated in brackets. Double asterisks indicate pD_*ap2g-hamSc*

donor plasmid integration downstream of the tagged *pfap2-g* gene. kb, kilo base; bp, base pairs; WT, wild type.

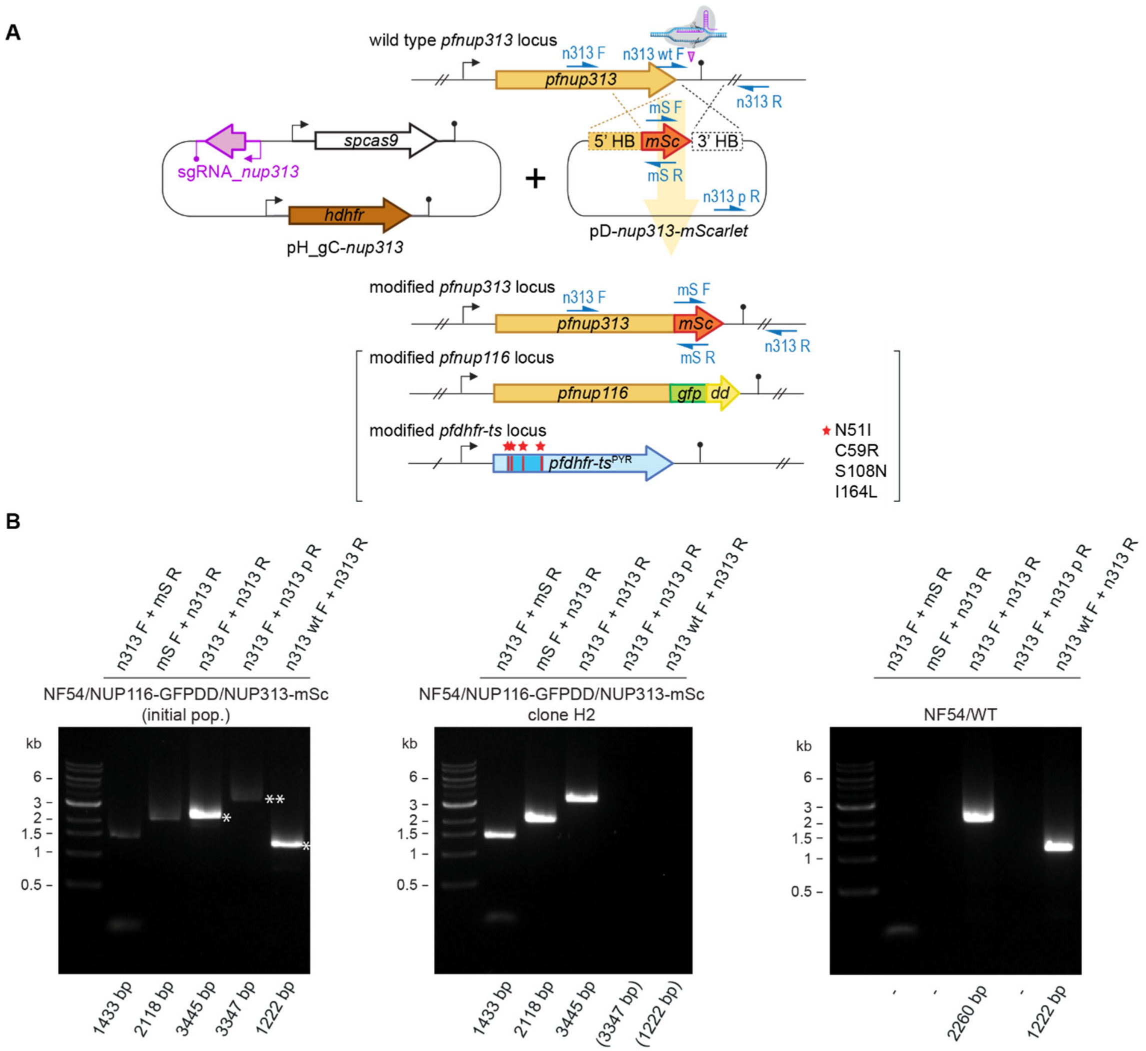

Figure S6. Cloning strategy and validation of NF54/NUP116-GFPDD/NUP313-mSc cell line. (A) Schematic illustration of the wild-type and modified *pfnup313* loci, alongside the plasmids (pH_gC-*nup313* and pD-*nup313-mScarlet*) used for iterative editing of the NF54/NUP116-GFPDD parasite line. Selection of gene-edited parasites was facilitated using the h*dhfr* drug resistance marker combined with WR99210-based selection. The previously modified *pfnup116-gfpdd* and *pfdhfr-ts*^{PYR} loci are shown below for reference. Names and relative positions of primers used for diagnostic PCRs are indicated by horizontal arrows. HB, homology box. **(B)** PCRs performed on the *pfnup313* locus on genomic DNA extracted from the initially selected transgenic NF54/NUP116-GFPDD/NUP313-mSc population (initial pop.) (left), NF54/NUP116-GFPDD/NUP313-mSc clone H2 (middle) and NF54 wild type control parasites (right). Names of primer pairs used for diagnostic PCRs are indicated on top. Expected DNA fragment sizes are given below gel images, with non-amplified fragments indicated in brackets. The single and double asterisks mark the presence of the unedited *pfnup313* target locus and pD_*nup313-mScarlet* donor plasmid integration downstream of the tagged *pfnup313* gene, respectively, in the initially selected transgenic population. kb, kilo base; bp, base pairs; WT, wild type.

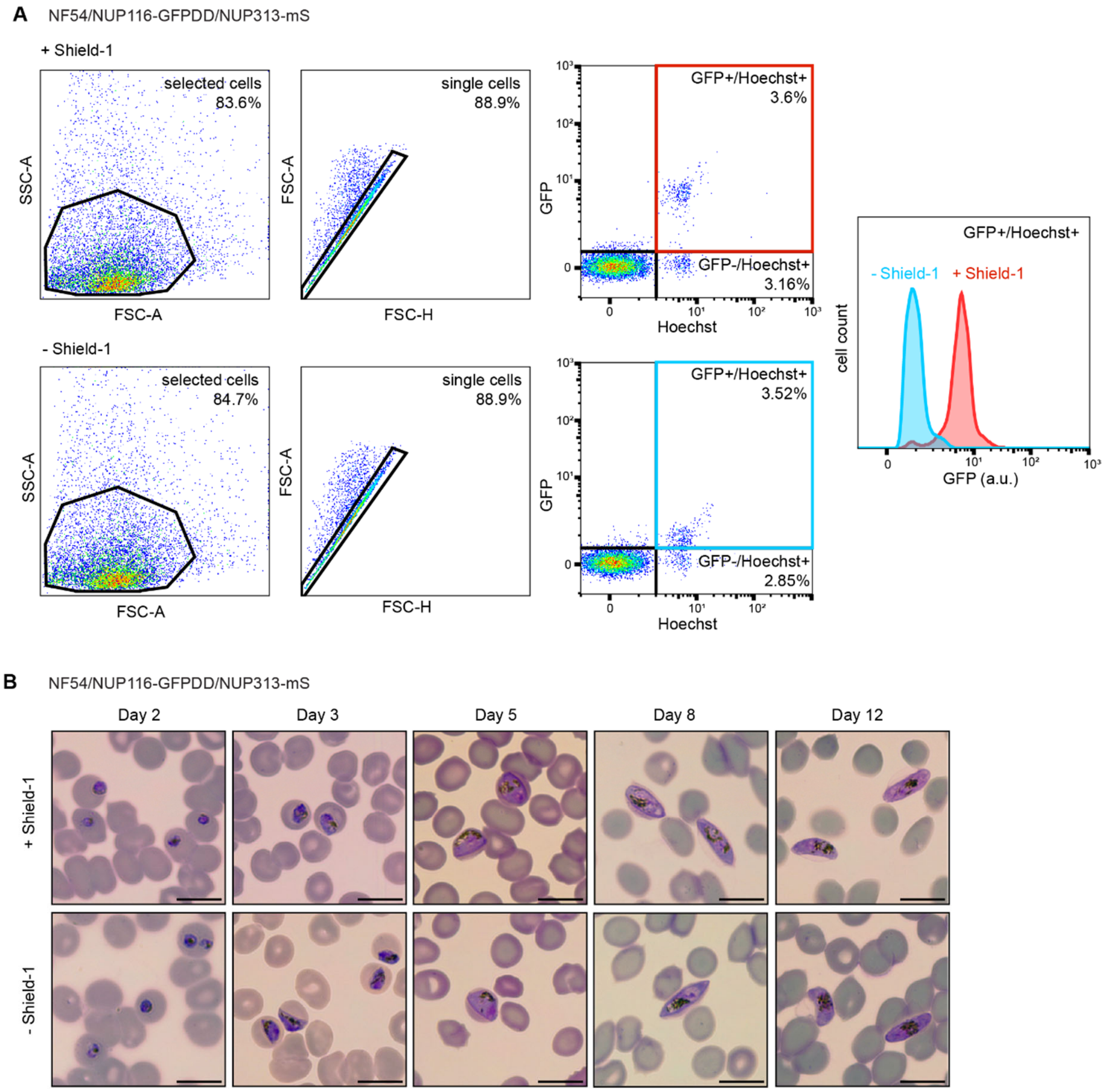

Figure S7. Phenotypic characterization of NF54/PfNUP116-GFPDD/PfNUP313-mSc gametocytes. (A) Flow cytometry-based quantification of GFP signal intensities in the progeny of NF54/PfNUP116-GFPDD/PfNUP313-mSc parasites induced for sexual commitment in the preceding cycle and grown in the presence (top) or absence (bottom) of Shield-1. Hierarchical gating was used to identify cytometry events corresponding to cells (FSC-A vs. SSC-A; left plot), singlets (FSC-A vs. FSC-H; middle plot) and infected RBCs (Hoechst+). Infected RBCs were gated into GFP+ and GFP– populations (right plot), with the GFP+/Hoechst+ and GFP–/Hoechst+ populations representing early stage I gametocytes (day 2 of gametocytogenesis) and asexual parasites, respectively. The rightmost plot compares the GFP fluorescence intensities in stage I gametocytes cultured in the presence (red) or absence (blue) of Shield-1. FSC, forward scatter. SSC, side scatter. A, area. H, height. **(B)** Representative images from Hemacolor-stained thin blood smears showing gametocyte development of NF54/PfNUP116-GFPDD/PfNUP313-mSc parasites cultured in the presence or absence of Shield-1. Scale bar, 10 µm.

Table S1. Quantification of PfNUP116-GFP and PfAP2-G-HAmSc expression by high content imaging

**replicate 1**									
**culture**									
mFA + CC	# total iRBCs (Hoechst+) 10424					% NUP116-expressing iRBCs 2.74			
	# asexual ring stages (AP2-G−)		# sexual ring stages (AP2-G+)		SCR (%)	% asexual ring stages (AP2-G−)		% sexual ring stages (AP2-G+)	
	NUP116−	NUP116+	NUP116−	NUP116+		NUP116+	NUP116−	NUP116+	NUP116−
	10036	67	102	219	3.08	0.66	99.34	68.22	31.78
mFA	# total iRBCs (Hoechst+) 6996					% NUP116-expressing iRBCs 18.08			
	# asexual ring stages (AP2-G−)		# sexual ring stages (AP2-G+)		SCR (%)	% asexual ring stages (AP2-G−)		% sexual ring stages (AP2-G+)	
	NUP116−	NUP116+	NUP116−	NUP116+		NUP116+	NUP116−	NUP116+	NUP116−
	5404	74	327	1191	21.70	1.35	98.65	78.46	21.54
**replicate 2**									
**culture**									
mFA + CC	# total iRBCs (Hoechst+) 13059					% NUP116-expressing iRBCs 3.97			
	# asexual ring stages (AP2-G−)		# sexual ring stages (AP2-G+)		SCR (%)	% asexual ring stages (AP2-G−)		% sexual ring stages (AP2-G+)	
	NUP116−	NUP116+	NUP116−	NUP116+		NUP116+	NUP116−	NUP116+	NUP116−
	12446	110	94	409	3.85	0.88	99.12	81.31	18.69
mFA	# total iRBCs (Hoechst+) 10625					% NUP116-expressing iRBCs 32.47			
	# asexual ring stages (AP2-G−)		# sexual ring stages (AP2-G+)		SCR (%)	% asexual ring stages (AP2-G−)		% sexual ring stages (AP2-G+)	
	NUP116−	NUP116+	NUP116−	NUP116+		NUP116+	NUP116−	NUP116+	NUP116−
	6838	213	337	3237	33.64	3.02	96.98	90.57	9.43
**replicate 3**									
**culture**									
mFA + CC	# total iRBCs (Hoechst+) 16023					% NUP116-expressing iRBCs 4.49			
	# asexual ring stages (AP2-G−)		# sexual ring stages (AP2-G+)		SCR (%)	% asexual ring stages (AP2-G−)		% sexual ring stages (AP2-G+)	
	NUP116−	NUP116+	NUP116−	NUP116+		NUP116+	NUP116−	NUP116+	NUP116−
	15229	113	74	607	4.25	0.74	99.26	89.13	10.87
mFA	# total iRBCs (Hoechst+) 9820					% NUP116-expressing iRBCs 30.38			
	# asexual ring stages (AP2-G−)		# sexual ring stages (AP2-G+)		SCR (%)	% asexual ring stages (AP2-G−)		% sexual ring stages (AP2-G+)	
	NUP116−	NUP116+	NUP116−	NUP116+		NUP116+	NUP116−	NUP116+	NUP116−
	6592	148	245	2835	31.36	2.20	97.80	92.05	7.95
**mean values**					SCR (%)	% asexual ring stages (AP2-G−)		% sexual ring stages (AP2-G+)	
**culture**						NUP116+	NUP116−	NUP116+	NUP116−
mFA + CC				**mean**	**3.73**	**0.76**	**99.24**	**79.56**	**20.44**
				stdev	0.60	0.11	0.11	10.56	10.56
mFA				**mean**	**28.90**	**2.19**	**97.81**	**87.02**	**12.98**
				stdev	6.34	0.84	0.84	7.46	7.46

Table S2. Oligonucleotides used in this study.

Note: restriction enzyme recognition sites or compatible single-stranded overhangs are highlighted in red.

Oligo name	Sequence (5' → 3')	Purpose	Plasmid/cell line/PCR product
AO_dhfr-ts_5'_F	CGTTGGCCGATTCATTAATCT CATTCCATTTATATAAGTATG ATTAGATTTG	cloning	p_gD-*dhfr*[pyrR]
dhfr-ts_5'_R	GTCTAAACTTATACATTTCCA TGGTAATACTCC	cloning	p_gD-*dhfr*[pyrR]
dhfr-ts_rec_F1	CCATGGAAATGTATAAGTTTA GACATGAAATATTTCAG	cloning	p_gD-*dhfr*[pyrR]
dhfr-ts_rec_R1	CCCAATTTGTACGTCCCATAA CTACAACG	cloning	p_gD-*dhfr*[pyrR]
dhfr-ts_rec_F2	CGTTGTAGTTATGGGACGTAC AAATTGGGAATC	cloning	p_gD-*dhfr*[pyrR]
dhfr-ts_rec_R2	CGGAACCTCCTAAAATGAAAC ATTTATAATAGTTC	cloning	p_gD-*dhfr*[pyrR]
dhfr-ts_3'_F	GTTTCATTTTAGGAGGTTCCG TTGTTTATC	cloning	p_gD-*dhfr*[pyrR]
AO_dhfr-ts_3'_R	CCTCTTCGCTATTACGCCAGA ACACCTACTCCCGTTC	cloning	p_gD-*dhfr*[pyrR]
PCRA_F	CTGGCGTAATAGCGAAGAGG	cloning	p_gD-*dhfr*[pyrR]
PCRA_R	CATTAATGAATCGGCCAACG	cloning	p_gD-*dhfr*[pyrR]
BspHI_F	GCGAGTTCATGAGCGAGGAA GCGGAAGAGC	cloning	p_gD-*dhfr*[pyrR]
HindIII_R	GTGCAAGCTTCTTCGCTATTA CGCCAG	cloning	p_gD-*dhfr*[pyrR]
dhfr-ts_gRNA3_F	TATTTAGTTATGGGAAGAACA AGC	sgRNA	p_gD-*dhfr*[pyrR]
dhfr-ts_gRNA3_R	AAACGCTTGTTCTTCCCATAA CTA	sgRNA	p_gD-*dhfr*[pyrR]
PCRA_F	CTGGCGTAATAGCGAAGAGG	cloning	pD-*gfpdd*_-BsaI
PCRA_R	CATTAATGAATCGGCCAACG	cloning	pD-*gfpdd*_-BsaI
gfpdd_F1	cgttggccgattcattaatgTCCAGTGGA atgagtaaaggag	cloning	pD-*gfpdd*_-BsaI
gfpdd_R1	accatgtgAtcCctcttttcgt	cloning	pD-*gfpdd*_-BsaI
gfpdd_F2	acgaaaagagGgaTcacatggt	cloning	pD-*gfpdd*_-BsaI
gfpdd_R2	cctcttcgctattacgccagtcattccagttttagaa gctccac	cloning	pD-*gfpdd*_-BsaI
gex1_5'_F	TCAGTGAGCGAGGAAGCGGAt gtatacattttctcatcagttagc	cloning	p_gCD-*gex1-gfp*
gex1_5'_R	cctttactcatTCCACTGGAaaaactatcta gtgaataatatttgtttaac	cloning	p_gCD-*gex1-gfp*
gex1_gfp_F	attattcactagatagttttTCCAGTGGAat gagtaaaggag	cloning	p_gCD-*gex1-gfp*
gex1_gfp_R	TTTAATCTATTATTAAATAAA TTTAtttgtatagttcatccatgccatg	cloning	p_gCD-*gex1-gfp*
gex1_hrp2_F	gcatggatgaactatacaaaTAAATTTAT TTAATAATAGATTAAAAATAT TATAAAAAT	cloning	p_gCD-*gex1-gfp*
gex1_hrp2_R	ggaaaaaagaaaacttaaaaTTTAATAA ATATGTTCTTATATATAATGA G	cloning	p_gCD-*gex1-gfp*
gex1_3'_F	ATAAGAACATATTTATTAAAttt taagttttctttttcccttttttgtg	cloning	p_gCD-*gex1-gfp*
gex1_3'_R	CTTCAGGGTAGCTGATATCGac agcactaagaagaatattcttcc	cloning	p_gCD-*gex1-gfp*
gex1_gRNA9_F	TATTtaaaggttaaatattcctta	sgRNA	sgRNA_gex1
gex1_gRNA9_R	AAACtaaggaatatttaacctta	sgRNA	sgRNA_gex1

nup116_5'_rec_F	GTCAGTGAGCGAGGAAGCGG Acccacaatgctagagaagg	cloning	p_gCD-*nup116-gfpdd*
nup116_5'_rec_R	cctttactcatTCCACTGGAAatttgAac attGgtttcTacCtc	cloning	p_gCD-*nup116-gfpdd*
nup116_gfpdd_F	tAgaaacCaatgtTcaaatTTCCAGTG GAatgagtaaaggag	cloning	p_gCD-*nup116-gfpdd*
nup116_gfpdd_R	ggcgattaattaatgtatatatttcattccagttttag aagctccac	cloning	p_gCD-*nup116-gfpdd*
nup116_3'_F	agcttctaaaactggaatgaaatatatacattaatt aatcgccttattc	cloning	p_gCD-*nup116-gfpdd*
nup116_3'_R	GACTTTTCTTCTTCAGGGTAG CTGATATCGaaagttgagcaattcctgttt tc	cloning	p_gCD-*nup116-gfpdd*
nup116_gRNA1_F	TATTaaattcattgatgcatcagt	sgRNA	p_gCD-*nup116-gfpdd*
nup116_gRNA1_R	AAACactgatgcatcaatgaattt	sgRNA	p_gCD-*nup116-gfpdd*
nup313_gRNA_F	TATTgcactttgtagagataagta	sgRNA	pHF_gC-*nup313*
nup313_gRNA_R	AAACtacttatctctacaaagtgc	sgRNA	pHF_gC-*nup313*
ha_F	ggggaaacaacaggaatattGGTtacccgtac gacgtccc	cloning	pD-*ap2-g-hamSc*
ha_R	CTGCTTCACCTTTACTACCTGC GGAggcataatctggaacatcg	cloning	pD-*ap2-g-hamSc*
d_F	ctattcacatatccatatgatatagg	PCR on gDNA	NF54/NUP116-GFPDD, NF54/GEX1-GFP, NF54/NUP116-GFPDD_NUP313-mSc, NF54/NUP116-GFPDD_AP2-G-HAmSc
d_R	agtaccattagcttcccatatcc	PCR on gDNA	NF54/NUP116-GFPDD, NF54/GEX1-GFP, NF54/NUP116-GFPDD_NUP313-mSc, NF54/NUP116-GFPDD_AP2-G-HAmSc
dm_F	acagtagacaacgttaacgac	PCR on gDNA	NF54/NUP116-GFPDD, NF54/GEX1-GFP, NF54/NUP116-GFPDD_NUP313-mSc, NF54/NUP116-GFPDD_AP2-G-HAmSc
dm_R	atgtcgttaacgttgtctactg	PCR on gDNA	NF54/NUP116-GFPDD, NF54/GEX1-GFP, NF54/NUP116-GFPDD_NUP313-mSc, NF54/NUP116-GFPDD_AP2-G-HAmSc
dw_F	actgtggataatgtaaatgatatgcc	PCR on gDNA	NF54/NUP116-GFPDD, NF54/GEX1-GFP, NF54/NUP116-GFPDD_NUP313-mSc, NF54/NUP116-GFPDD_AP2-G-HAmSc
dw_R	ggcatatcatttacattatccacag	PCR on gDNA	NF54/NUP116-GFPDD, NF54/GEX1-GFP, NF54/NUP116-GFPDD_NUP313-mSc, NF54/NUP116-GFPDD_AP2-G-HAmSc
n116_F	atgaaaagaaaaagaggaagagaag	PCR on gDNA	NF54/NUP116-GFPDD
n116_R	cttcccatttgatgctgcc	PCR on gDNA	NF54/NUP116-GFPDD
gex1_F	cacagcaatatcaaaataaaattaagg	PCR on gDNA	NF54/GEX1-GFP
gex1_R	tatcaaacgaaaaaaaaggaggg	PCR on gDNA	NF54/GEX1-GFP
gfp_F	acatggcatggatgaactatacaaa	PCR on gDNA	NF54/NUP116-GFPDD, NF54/GEX1-GFP
gfp_R	tccagtgaaaagttcttctcct	PCR on gDNA	NF54/NUP116-GFPDD, NF54/GEX1-GFP
p_gCD_R	attgagcagaggatatgcg	PCR on gDNA	NF54/NUP116-GFPDD, NF54/GEX1-GFP
n313_F	tgagcatatagtaccatcagaatgg	PCR on gDNA	NF54/NUP116-GFPDD_NUP313-mSc
n313_R	gatacaagggaaggaatacaacg	PCR on gDNA	NF54/NUP116-GFPDD_NUP313-mSc
n313_p_R	tgtcggggctggcttaac	PCR on gDNA	NF54/NUP116-GFPDD_NUP313-mSc
n313_wt_F	acacaataaaatgttcacggaataatg	PCR on gDNA	NF54/NUP116-GFPDD_NUP313-mSc
ap2-gF	cattatgtatcagcaaatgcag	PCR on gDNA	NF54/NUP116-GFPDD_AP2-G-HAmSc
ap2-gR	gtacaacaaaaactgaactc	PCR on gDNA	NF54/NUP116-GFPDD_AP2-G-HAmSc
AO_Seq_F	gcgaggaagcggaagagc	PCR on gDNA	NF54/NUP116-GFPDD_AP2-G-HAmSc
mS_F	ggaggtgcagttacagtaacacaag	PCR on gDNA	NF54/NUP116-GFPDD_NUP313-mSc, NF54/NUP116-GFPDD_AP2-G-HAmSc
mS_R	gcattactggtccatctggtgga	PCR on gDNA	NF54/NUP116-GFPDD_NUP313-mSc, NF54/NUP116-GFPDD_AP2-G-HAmSc
d_SeqF	gccatatgtgcatgttgtaagg	Sanger sequencing	d_F/d_R PCR product
